# Questing for homoleptic mononuclear manganese complexes with monodentate O-donor ligands†

**DOI:** 10.1039/d4sc00543k

**Published:** 2024-03-05

**Authors:** Alberto Pérez-Bitrián, Julen Munárriz, Konstantin B. Krause, Johanna Schlögl, Kurt F. Hoffmann, Johanna S. Sturm, Amiera N. Hadi, Christian Teutloff, Anja Wiesner, Christian Limberg, Sebastian Riedel

**Affiliations:** a Fachbereich Biologie, Chemie, Pharmazie, Institut für Chemie und Biochemie − Anorganische Chemie, Freie Universität Berlin Fabeckstraße 34/36 Berlin 14195 Germany alberto.perez-bitrian@hu-berlin.de s.riedel@fu-berlin.de; b Institut für Chemie, Humboldt-Universität zu Berlin Brook-Taylor-Straße 2 Berlin 12489 Germany; c Departamento de Química Física and Instituto de Biocomputación y Física de Sistemas Complejos (BIFI), Universidad de Zaragoza Pedro Cerbuna 12 Zaragoza 50009 Spain julen@unizar.es; d Fachbereich Physik, Freie Universität Berlin Arnimallee 14 Berlin 14195 Germany

## Abstract

Compounds containing Mn–O bonds are of utmost importance in biological systems and catalytic processes. Nevertheless, mononuclear manganese complexes containing all O-donor ligands are still rare. Taking advantage of the low tendency of the pentafluoroorthotellurate ligand (teflate, OTeF_5_) to bridge metal centers, we have synthesized two homoleptic manganese complexes with monomeric structures and an all O-donor coordination sphere. The tetrahedrally distorted Mn^II^ anion, [Mn(OTeF_5_)_4_]^2−^, can be described as a high spin d^5^ complex (*S* = 5/2), as found experimentally (magnetic susceptibility measurements and EPR spectroscopy) and using theoretical calculations (DFT and CASSCF/NEVPT2). The high spin d^4^ electronic configuration (*S* = 2) of the Mn^III^ anion, [Mn(OTeF_5_)_5_]^2−^, was also determined experimentally and theoretically, and a square pyramidal geometry was found to be the most stable one for this complex. Finally, the bonding situation in both complexes was investigated by means of the Interacting Quantum Atoms (IQA) methodology and compared to that of hypothetical mononuclear fluoromanganates. Within each pair of [MnX_*n*_]^2−^ (*n* = 4, 5) species (X = OTeF_5_, F), the Mn–X interaction is found to be comparable, therefore proving that the similar electronic properties of the teflate and the fluoride are also responsible for the stabilization of these unique species.

## Abstract 2

High spin monomeric complexes [Mn^II^(OTeF_5_)_4_]^2−^ and [Mn^III^(OTeF_5_)_5_]^2−^ represent novel homoleptic coordination environments at Mn(ii) and Mn(iii) centers with monodentate O-donor ligands.

## Graphical abstract

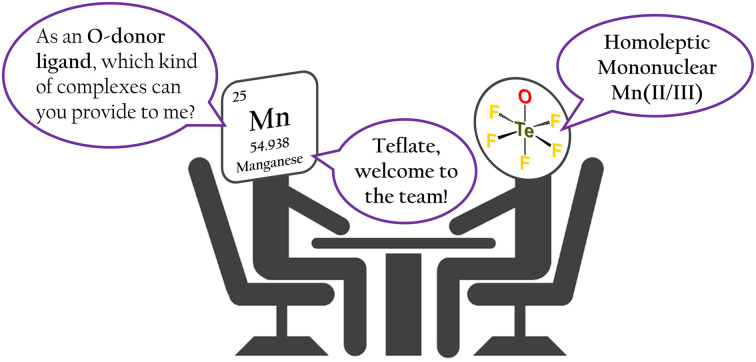

## Introduction

Manganese is a key element in biological systems,^1^ being particularly relevant in the photosynthesis,^2–6^ as well as in a diversity of catalytic processes.^7–13^ One of the facts that makes it especially interesting is the wide range of oxidation states that it can present, varying from −III to +VII.^14^ Whereas high oxidation states are stabilized by oxo ligands, as in the [MnO_4_]^−^ ion or in the binary Mn_2_O_7_, fluoride is only able to stabilize medium oxidation states.^15–17^ In fact, manganese fluorides are only known up to oxidation state +IV in MnF_4_ and in the related [MnF_6_]^2−^, although oxidation state +VII is attained in the oxyfluoride MnO_3_F.^18^

Compounds containing Mn–O bonds are involved in catalytic and enzymatic reactions.^19–22^ Notably, the chemistry of manganese complexes with oxygen ligands is mainly dominated by polymetallic species, including oxo ligands in the higher oxidation states, whereas alkoxides or carboxylates are the preferred ligands for lower oxidation states.^14,23,24^ To prevent aggregation and enable the formation of mononuclear complexes, bulky alkoxide ligands, as well as fluorinated ones, constitute suitable ligand scaffolds.^25,26^ In this regard, the pentafluoroorthotellurate group (teflate, OTeF_5_) also offers unique possibilities, as it provides an O-donor ligand system with a low tendency to bridge metal centers.^27,28^ Its electron-withdrawing properties, similar to those of fluoride, made us envision the possibility of using this monodentate ligand for the synthesis of unprecedented homoleptic mononuclear manganese compounds containing Mn–O bonds, which would be analogues of the well-studied low-valent fluoromanganates.^18^ Here, we report the synthesis of two different manganese teflate complexes in oxidation states +II and +III, *i.e.*, [Mn(OTeF_5_)_4_]^2−^ and [Mn(OTeF_5_)_5_]^2−^, and the investigation of their structural and electronic properties by means of a combined experimental and theoretical approach.

## Results and discussion

### Synthesis and characterization of [NEt_4_]_2_[Mn(OTeF_5_)_4_]

**Scheme 1:**
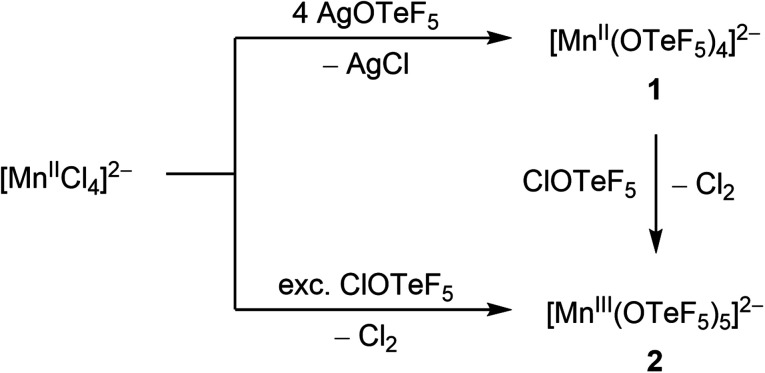
Synthetic routes to complexes [Mn(OTeF_5_)_4_]^2−^ and [Mn(OTeF_5_)_5_]^2−^. The cation is [NEt_4_]^+^ in all cases.

The manganese(ii) compound [NEt_4_]_2_[Mn(OTeF_5_)_4_] (1) can be synthesized by reacting [NEt_4_]_2_[MnCl_4_] with AgOTeF_5_ in CH_2_Cl_2_ and after removal of the formed AgCl *via* filtration it is isolated as an off-white solid (Scheme 1). The product exhibits a similar IR spectrum to the related [NEt_4_]_2_[M(OTeF_5_)_4_] compounds (M = Ni, Co, Fig. S2†),^29,30^ indicating the four-coordinate nature of the manganate anion. The Te–O vibration observed at 854 cm^−1^ signifies the ionic nature of the Mn–OTeF_5_ bond.^31^

**Fig. 1:**
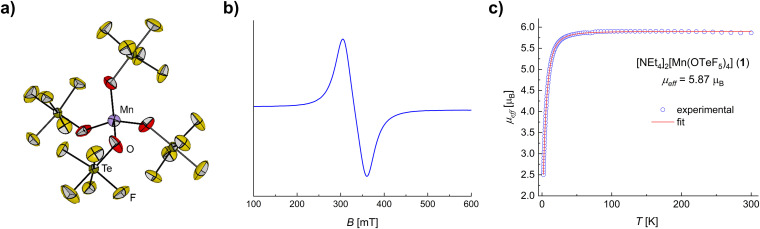
(a) Molecular structure of the [Mn(OTeF_5_)_4_]^2−^ anion in the solid state as found in crystals of 1*. The [PPh_4_]^+^ cations have been omitted for clarity. Displacement ellipsoids are set at 50% probability. Selected bond lengths [pm] and angles [°]: Mn–O 202.1(3), O–Mn–O 112.23(11)/104.1(2). For crystallographic details see the ESI.† (b) X-band EPR spectrum of 1 in DCM (5.0 mM) at 293 K. The spectrum yields *g*_eff_ = 2.02. The rather high *g*-value is attributed to the contribution of the zero-field splitting, and the unresolved lines to a yet unknown broadening mechanism. (c) Experimental *μ*_eff_*versus T* plot and fit for compound 1.

Despite numerous attempts under different conditions, only intergrown and highly twinned crystals of compound 1 could be obtained, which were not suitable for single-crystal X-ray diffraction. Gratifyingly, the use of a different cation, namely [PPh_4_]^+^, allowed the preparation and growth of single crystals of [PPh_4_]_2_[Mn(OTeF_5_)_4_] (1*). Compound 1* crystallizes in the tetragonal space group *I*4_1_/*a* (see the ESI† for details). The [Mn(OTeF_5_)_4_]^2−^ anion (Fig. 1a), which appears well separated from the [PPh_4_]^+^ cations, exhibits a distorted tetrahedral geometry, similar to those observed for the related [M(OTeF_5_)_4_]^2−^ anions (M = Ni, Co).^29,30^ Nevertheless, the distortion at the Mn(ii) center is much less pronounced in this case, with a geometry index of *τ*_4_ = 0.96.^32^

The combination of manganese and the teflate ligand was already known, yet only with manganese in oxidation state +I in compound [Mn(CO)_5_(OTeF_5_)].^33–35^ The uniqueness of the [Mn(OTeF_5_)_4_]^2−^ anion lies in the presence of four single Mn–O bonds, compound 1* actually entailing the first example of a homoleptic mononuclear Mn(ii) species with four monodentate O-donor ligands. A compound close to this situation is the coordination polymer [Mn(pyc)_4_(*μ*_2_-SO_4_)·H_2_O]_∞_ (pyc = 4-carboxy-1-methylpyridinium), where each Mn(ii) center is coordinated by four pyc ligands in an almost square planar arrangement, but these subunits are connected by *η*^4^,*μ*_2_-SO_4_ bridges.^36^ Noteworthily, in this context, compound [Mn^II^(pin^F^)_2_]^2−^ (pin^F^ = perfluoropinacolate) was recently reported, containing two chelating ligands.^37^ This compound exhibits a pseudotetrahedral geometry around the metal center with a much more prominent distortion (*τ*_4_ = 0.43) than the anion in compound 1* (Fig. 1a), probably because of the two chelating ligands. In our case, a perfectly tetrahedral geometry at the metal center should be expected, yet it is slightly distorted probably due to steric reasons. These structures are unusual, as most of the known manganese(ii) complexes with O-donors are heteroleptic, ranging from mono- and dinuclear coordination compounds to clusters of different sizes, or even polymeric chain structures.^14,23,24^ In this regard, also Mn(ii) complexes containing two alkoxido or aryloxido ligands and additional solvent molecules are known.^38–40^ In our case, the use of a non-donor solvent has further helped isolate the homoleptic species, which in conjunction with the low tendency of the teflate to bridge metal centers gives rise to the monomeric nature of the compound.

The existence of a Mn(ii) center within the anion [Mn(OTeF_5_)_4_]^2−^ (see the ESI† for bond valence sum analyses) was confirmed *via* electron paramagnetic resonance (EPR) spectroscopy. The X-band EPR spectrum of 1 recorded at room temperature in CH_2_Cl_2_ (Fig. 1b) does not show six distinct lines, as would be expected for an *I* = 5/2 nucleus. Only the corresponding W-band spectrum (Fig. S10†) reveals the expected hyperfine splitting and gives *g*_*iso*_ = 2.000 (*A*(^55^Mn) = 255 MHz), as expected for a high spin (HS), mononuclear Mn(ii) complex.^41^ We attribute the unresolved hyperfine splitting at the X-band to a yet unknown broadening mechanism involving the metal center and the [OTeF_5_]^−^ ligand.

Due to the stabilization of the half-filled d shell and the low charge of the metal center, typically Mn(ii) complexes exhibit a HS configuration (*S* = 5/2),^42^ Mn(ii) centers in low spin (LS) configurations being very scarce.^14,23^ Additionally, because of the weak/medium-field character of the teflate ligand, a HS is expected for 1 all the more.^29^ In line with this, 1 was determined to have an effective magnetic moment of *μ*_eff_ = 5.87 *μ*_B_ (Fig. 1c), being very close to the spin-only value of a d^5^ HS system, *i.e.*, *μ*_s.o._ = 5.92 *μ*_B_. This excellent agreement can be explained by the lack of orbital contribution to the magnetic moment due to the HS electronic configuration leading to a ^6^S ground term,^43^ similarly to the [MnX_4_]^2−^ anions (X = Cl, Br, I).^44^ The magnetic data were successfully simulated, as shown in Fig. 1c and the fit parameters for 1 are reported in the ESI.† Typically the magnetic anisotropy of a HS Mn(ii) center is characterized by a small zero-field splitting (corresponding *D* values < 1 cm^−1^)^45^ and indeed a *D* = 0.62 cm^−1^ was inferred for 1.

To further understand the nature of the [Mn(OTeF_5_)_4_]^2−^ anion, we investigated its electronic structure by means of a theoretical study. First, we performed a DFT structure optimization of the sextet ground state by using B3LYP-D3BJ, M06, M06-L and TPPSh functionals. All of them provided similar geometries with structural parameters *τ*_4_ = 0.99 (B3LYP-D3BJ, M06 and TPPSh) or *τ*_4_ = 0.98 (M06-L), which are in good agreement with the experimental one (*vide supra*). Given the problems that might arise when studying the electronic structure of first–row transition metals by means of DFT,^46–49^ we also applied multi-reference calculations. Namely, on the B3LYP-D3BJ-optimized structure, we combined the state-average complete active space self-consistent field (SA-CASSCF) that accounts for static electron correlation,^50^ with *n*-electron valence state perturbation theory (NEVPT2)^51–53^ to account for dynamic electron correlation. In the active space, we incorporated the Mn–O bonding orbitals based on the 3d orbitals, along with the primarily 3d orbitals of the metal and the corresponding 4d orbitals, to accurately consider the effects of double-d shell correlation.^54–56^ As a result, we obtained an active space composed of 11 electrons in 13 molecular orbitals, SA-CASSCF(11,13)/NEVPT2 (Fig. S11†). As anticipated, the ground state corresponds to the sextet based on the ^6^A_1_ term, where the five 3d orbitals of the manganese are partially occupied. Note that this configuration has a weight of 98.8%, which allows for the consideration that the sextet ground state has a prominent single-reference character. The first excited state would correspond to a quartet that lies 286 kJ mol^−1^ above in energy (see Table S6† for the full set of states). Overall, this picture justifies the use of DFT.

### Synthesis and characterization of [NEt_4_]_2_[Mn(OTeF_5_)_5_]

The reaction of [NEt_4_]_2_[MnCl_4_] with ClOTeF_5_ takes place with oxidation of the Mn(ii) center to Mn(iii) and coordination of five teflate ligands to the metal center (Scheme 1). Compound [NEt_4_]_2_[Mn(OTeF_5_)_5_] (2) is selectively formed as a deep blue solid, whereas Cl_2_ is released as a yellow gas. The role of ClOTeF_5_ as an oxidizer towards various metals has been previously reported, *e.g.* in the preparation of [Mo(OTeF_5_)_6_]^57^ or [ReO(OTeF_5_)_4_].^58^ This behavior is in contrast with the synthesis of the related [NEt_4_]_2_[M^II^(OTeF_5_)_4_] salts (M = Ni, Co), in which the oxidation state and coordination number of the metals remain unaltered upon reaction of the corresponding [NEt_4_]_2_[M^II^Cl_4_] salt with neat ClOTeF_5_.^29,30^ Interestingly, compound 2 can also be obtained by oxidation of 1 with ClOTeF_5_, which is advantageous, as a much lower amount of hypochlorite is needed. In this case, the IR spectrum (see Fig. S4†) exhibits a broad band at 827 cm^−1^ for the Te–O vibrations, denoting the ionic nature of the Mn–OTeF_5_ bond also in the Mn(iii) species.^31^

**Fig. 2:**
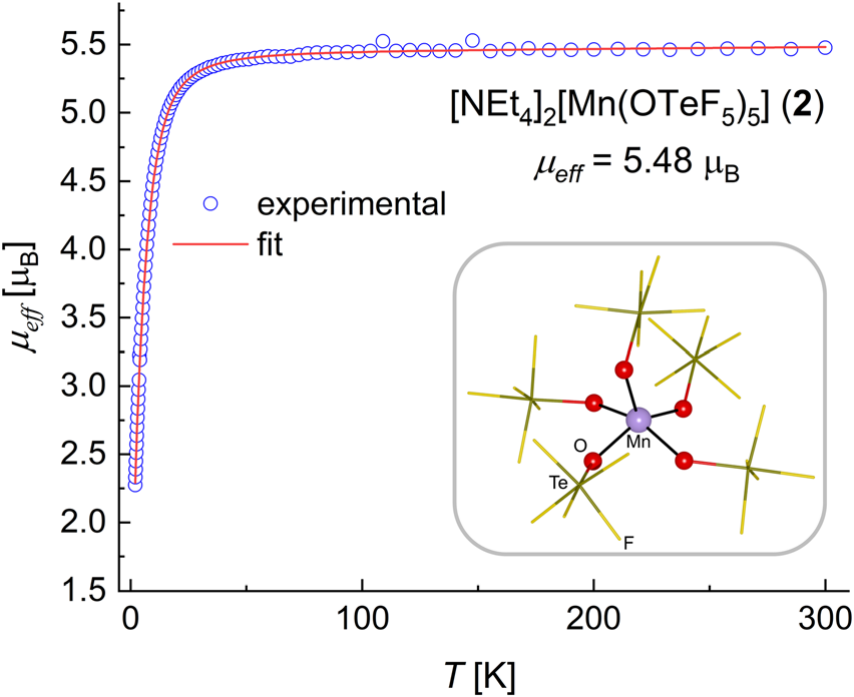
Experimental *μ*_eff_*versus T* plot and fit for compound 2. A schematic representation of the [Mn(OTeF_5_)_5_]^2−^ anion in the solid state is shown inside the frame, with the first coordination sphere of the Mn(iii) center highlighted.

All attempts to prepare crystals of 2 proved to be of an overall low quality, resulting in an aggregation of different components that made it difficult to treat the structure as a non-merohedral twin, additionally characterized by severe disorder. Unfortunately, although the use of the [PPh_4_]^+^ cation seemed promising after the successful crystallization of 1*, this cation proved to be unstable in the presence of ClOTeF_5_. Therefore, only the connectivity in compound 2 could be established from our crystallographic analysis. Compound 2 consists of two [NEt_4_]^+^ cations and the [Mn(OTeF_5_)_5_]^2−^ anion, without any significant interaction among them. Interestingly, the [Mn(OTeF_5_)_5_]^2−^ anion shows a monomeric structure with a {MnO_5_} core displaying an overall square pyramidal geometry (Fig. 2). Such an isolated core in a homoleptic complex with monodentate ligands is without precedence in the literature: the vast majority of species containing {MnO_5_} cores are clusters and polynuclear species, including both homo- and heterometallic compounds. Mononuclear representatives contain either chelating ligands^37,59–65^ or solvent molecules^66,67^ completing the coordination sphere, yet none of them has the same O-donor ligand occupying the five coordination sites around the manganese center.

An effective magnetic moment *μ*_eff_ = 5.48 *μ*_B_ was determined for compound 2 (Fig. 2). This is higher than the spin-only value of *μ*_s.o._ = 4.90 *μ*_B_ expected for four unpaired electrons at a Mn(III) center (*S* = 2) and also than the value found in the structurally related^68–70^ [MnCl_5_]^2−^ anion.^71–73^ Nevertheless, it is comparable with the magnetic moments determined for other square pyramidal dianionic {Mn^III^O_5_} complexes,^37^ and it clearly demonstrates a high-spin configuration that is in line with the weak/medium-field character of the teflate ligand.^29^ The fact that 2 contains a Mn center in the oxidation state +III was further confirmed by the results of a bond valence sum analysis (see the ESI†).

**Fig. 3:**
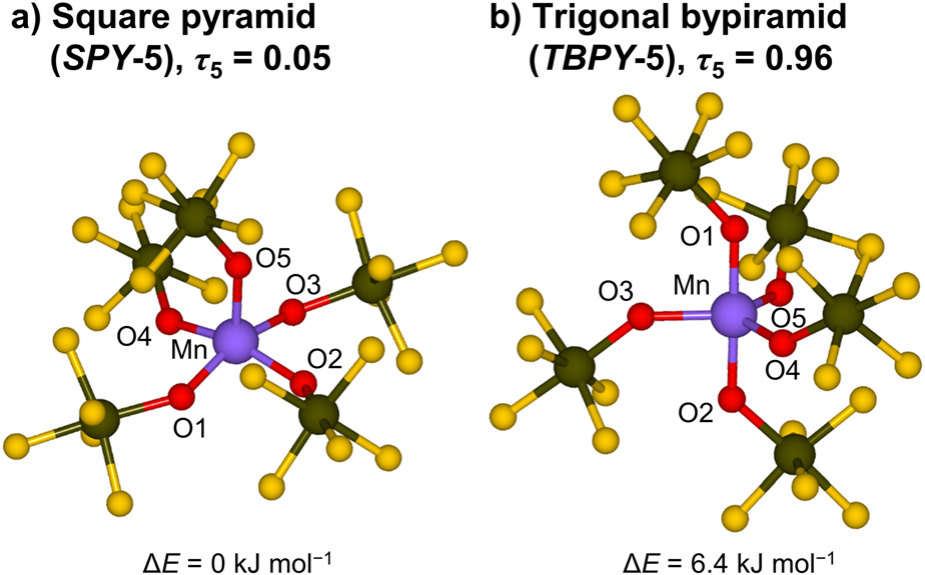
DFT structures of the [Mn(OTeF_5_)_5_]^2−^ anion optimized at the B3LYP-D3BJ level of theory and calculated relative energies. (a) Square pyramidal structure. Selected bond lengths [pm]: Mn–O1 191.07, Mn–O2 190.79, Mn–O3 191.18, Mn–O4 190.01, and Mn–O5 201.41. (b) Trigonal bipyramidal structure, obtained after imposition of geometrical constraints. Selected bond lengths [pm]: Mn–O1 188.60, Mn–O2 188.15, Mn–O3 196.00, Mn–O4 196.38, and Mn–O5 197.36.

To gain further insights into the geometry of the [Mn(OTeF_5_)_5_]^2−^ anion beyond the limitations of our crystallographic data, we undertook a theoretical analysis. As five-coordinate transition-metal complexes can exhibit two different geometries: square pyramidal (SPY-5) and trigonal bipyramidal (TBPY-5),^74^ we considered both structural possibilities for our calculations. All the optimizations converged to the experimentally observed square pyramid, regardless of the starting structure. Interestingly, we found two intimately related, although slightly different, SPY-5 structures, whose energy difference is lower than 1.0 kJ mol^−1^ for the B3LYP-based methodology. In order to obtain a structure with the TBPY-5 geometry, geometrical constraints had to be imposed (see the ESI† for additional details). Nevertheless, the obtained TBPY-5 structure (for B3LYP-D3BJ) is almost isoenergetic, being only 6.4 kJ mol^−1^ higher in energy. In general, the same trend is observed when using M06-L, M06 and TPSSh (Table S9†). Both optimized structures at B3LYP-D3BJ, with indicated bond lengths, calculated geometry indices *τ*_5_,^75^ and relative energies are shown in Fig. 3.

Furthermore, although the HS d^4^ electronic configuration of our system is the most common one for the electronic ground state of a Mn(iii) center,^42^ the existence of complexes exhibiting spin crossover (SCO) that involve an intermediate spin (IS) configuration (*S* = 1, two unpaired electrons) or even with the LS configuration (*S* = 0) has been reported for some systems.^76–84^ Therefore, despite SCO not being experimentally observed for compound 2, we optimized the structure at the DFT level (by using the same functionals as for the Mn(ii) species), considering not only the HS state as described above but also the IS and LS states. While there was significant dispersion in the relative energies of the various spin states, all the functionals revealed a HS quintet ground state. The energy difference to the IS state is in the range 94.7–188.5 kJ mol^−1^ (depending on the functional), being 94.7 kJ mol^−1^ for TPSSh, which has generally been ranked as a suitable choice for the study of the electronic structure of Mn complexes.^47,80,85^ Note that this value is close to the B3LYP one, which is 112.8 kJ mol^−1^.

**Fig. 4:**
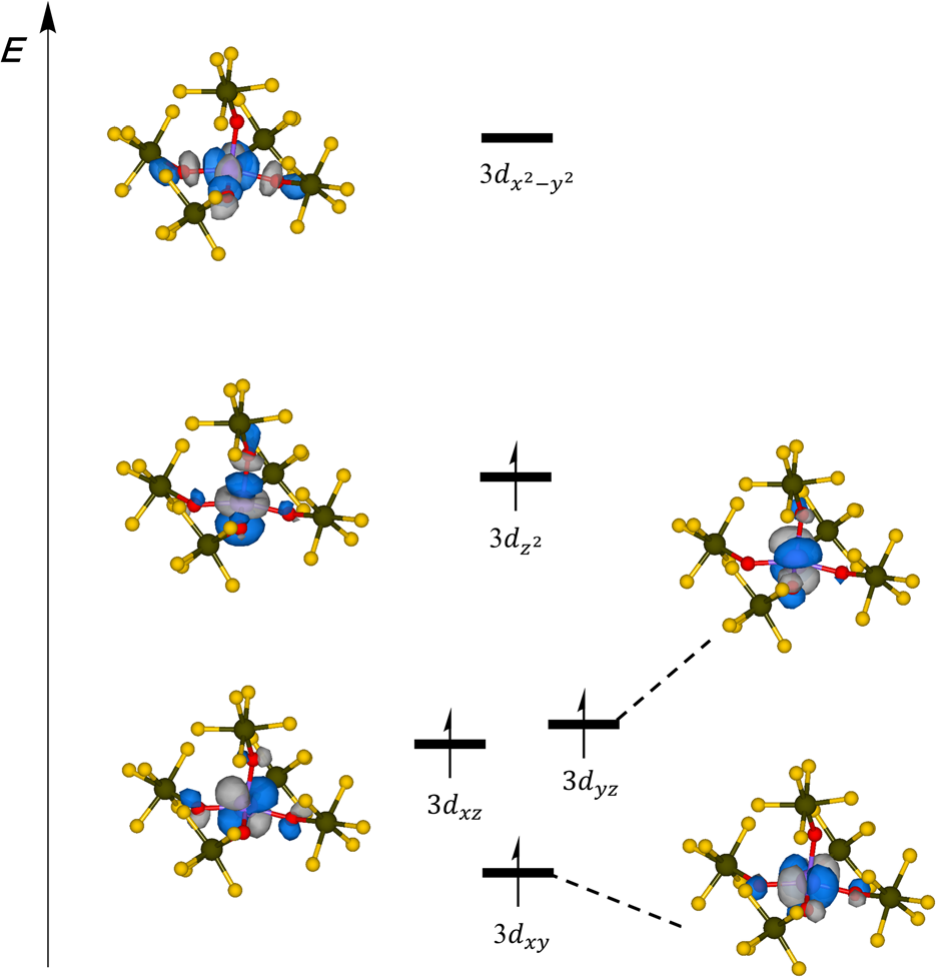
Schematic representation of the energy diagram of the SA-CASSCF(12,14) MOs mainly composed of Mn 3d orbitals for the [Mn(OTeF_5_)_5_]^2−^ anion.

As a further check of consistency we applied multi-reference SA-CASSCF(12,14)/NEVPT2 calculations on the global minimum structure. Consistent with the calculations performed for the [Mn(OTeF_5_)_4_]^2−^ anion, our active space comprised Mn–O bonding orbitals that exhibit substantial involvement of Mn 3d orbitals. Additionally, we considered the five 3d-based orbitals that prominently incorporate Mn and the corresponding 4d orbitals in order to account for the double-shell effect (Fig. S12†). According to the NEVPT2 calculations, a quintet ground state was determined, where the Mn 3d_*x*^2^−*y*^2^_ orbital is unoccupied (Fig. 4). This specific configuration holds a weight of 96.6%, indicating that the state can be described as a single-reference state in broad terms. All other states are provided in Table S7,† in which it can be seen that the lowest triplet state is 217.8 kJ mol^−1^ higher in energy, and this difference increases to 333.6 kJ mol^−1^ for the lowest singlet. The HS quintet state is also in agreement with the weak/medium-field character of the teflate ligand,^29^ as well as with our magnetic measurements (*vide supra*).

### Chemical bonding analyses

Compounds 1 and 2 represent unique examples of homoleptic mononuclear low-valent manganese compounds with all monodentate O-donor ligands. These features are enabled by the electronic similarities of the fluoride and teflate ligands, together with the limited tendency of the teflate to bridge metal centers.^27,28^ In fact, when compared to the corresponding fluoride analogues, it is the latter reason that hinders the formation of extended structures in the solid state, contrary to [MnF_4_]^2−^, which exists as layers, or [MnF_5_]^2−^, which forms chains.^18^

With the aim of comparing the bonding mode of such electronically similar compounds, namely [Mn(OTeF_5_)_*n*_]^2−^ (*n* = 4, 5) and the corresponding hypothetical monomeric [MnF_*n*_]^2−^ (*n* = 4, 5), we undertook a bonding analysis by means of the Interacting Quantum Atoms (IQA) energy decomposition scheme,^86^ which we have previously applied to related [CoX_4_]^2−^ complexes (X = OTeF_5_, F, Cl).^30^ IQA is an orbital-invariant and parameter-free approach that applies a scalar topological partition to divide the space into regions associated with chemically meaningful entities. As it is customarily performed, we coupled IQA with the partition of space provided by the Quantum Theory of Atoms in Molecules (QTAIM).^87^ This way, the space is divided into different atoms. Within this framework, the total energy is divided into intra-atomic and inter-atomic contributions between pairs of atoms or groups of atoms (say A and B). The latter term (*E*^AB^_inter_) can be further decomposed into a classical electrostatic (*V*^AB^_cl_) and an exchange-correlation contribution (*V*^AB^_xc_), which is directly related to bond covalency.^88–90^ It should be noted that *V*^AB^_xc_ has also been proposed as a direct measure of bond strength, as the classical (Coulomb) interaction is significantly affected by long–range interactions between highly charged groups.^91^ Herein, we considered the interaction between the manganese center and a given group (X) that might be a single atom, as in the case of F in [MnF_*n*_]^2−^, or the combination of various atoms, as the teflate ligand in [Mn(OTeF_5_)_*n*_]^2−^. For the latter, its interaction with the metal is obtained by adding all the pairwise interactions between it and each of the atoms belonging to the group.

The classical (ionic) and exchange-correlation (covalent) interaction terms for [Mn(OTeF_5_)_4_]^2−^ and [MnF_4_]^2−^ are provided in Table 1. They are referred to as *V*^MX^_cl_ and *V*^MX^_xc_, as they account for the interaction between the metal center (M) and the ligand group (X). For comparison purposes, the Co analogues are also provided.^30^ The *V*^MX^_xc_ term for [Mn(OTeF_4_)_4_]^2−^ (−251.0 kJ mol^−1^) is comparable to that of [MnF_4_]^2−^ (−233.1 kJ mol^−1^), which is in line with the similar covalent character of both ligands. In this regard, it is noticeable that, albeit they are still quite similar, the difference between these terms (17.9 kJ mol^−1^) is smaller than that for the Co-based compounds (28.6 kJ mol^−1^), and that the covalent interaction in [Mn(OTeF_4_)_4_]^2−^ is significantly weaker than that for [Co(OTeF_4_)_4_]^2−^ (−251.0 *vs.* −293.3 kJ mol^−1^).

**Table 1:** Calculated M–X distance (pm, M = Mn, Co), *V*^MX^_cl_ (kJ mol^−1^), *V*^MX^_xc _(kJ mol^−1^), and QTAIM charges (|*e*^−^|) for [MX_4_]^2−^ complexes (M = Mn, Co; X = OTeF_5_, F)

[MX_4_]^2−^		*d* (M–X)	*V* ^MX^_cl_	*V* ^MX^_xc_	*q* (M)	*q* (X)
M	X					
Mn	OTeF_5_	202.43	−705.4	−251.0	1.58	−0.90
	F	203.69	−903.4	−233.1	1.54	−0.88
Co^a^	OTeF_5_	196.72	−617.4	−293.3	1.44	−0.86
	F	197.62	−842.8	−264.7	1.44	−0.86

a Values taken from ref. 30.

Electrostatic interactions also deserve a special comment. Given the lower electronegativity of Mn with respect to Co, it is somehow evident that the charge of the Mn center should be higher than that of Co, which is indeed an observed fact (see Table 1). In this line, the classical electrostatic M–OTeF_5_ interaction is more favorable for [Mn(OTeF_5_)_4_]^2−^ than for [Co(OTeF_5_)_4_]^2−^ (−705.4 *vs.* −617.4 kJ mol^−1^, respectively), which compensates for the decrease in the covalent interaction term.

We now consider the Mn(iii) anion. First, we checked if the structure of the hypothetical monomeric [MnF_5_]^2−^ anion is the same as for [Mn(OTeF_5_)_5_]^2−^. Surprisingly, the global minimum for [MnF_5_]^2−^ is represented by a TBPY-5 structure instead of the SPY-5 geometry of the [Mn(OTeF_5_)_5_]^2−^ anion. Nonetheless, the energy difference between both structures is, as in the case of the teflate compound, very small. Namely, the square pyramidal structure is, at the B3LYP-D3BJ level, 1.4 kJ mol^−1^ higher in electronic energy and exhibits an imaginary frequency of 9i cm^−1^ that corresponds to the transition to the TBPY-5 structure *via* Berry pseudorotation. Note that comparable results were obtained for the other functionals (see Table S13† and additional explanations provided in the ESI†).

With this in hand, we proceed to analyze the energetics of the Mn–OTeF_5_ interaction in the [Mn(OTeF_5_)_5_]^2−^ anion. The *V*^MnX^_xc_ term of the IQA framework for the SPY-5 structure is significantly larger (in absolute value) for the bond with the basal teflates than for the apical one (−330.3 and −248.8 kJ mol^−1^, respectively). This fact is somehow expected, as the Mn–O bond length with the apical oxygen in the SPY-5 structure (201.41 pm) is much longer than that with the basal oxygen atoms (190.76 pm av.), as can be seen in Fig. 3. In the same line, the interaction energy for the axial Mn–O bonds in the TBPY-5 structure is more favorable than that for the equatorial bonds (−352.6 and −287.4 kJ mol^−1^, respectively), as also anticipated from the shorter Mn–O bonds (Fig. 3) in the axial positions (188.38 pm av.) than in the equatorial ones (196.58 pm av.). When comparing the *V*^MnX^_xc_ term for both ligands (X = OTeF_5_, F), a similar covalent character of the interaction is observed in both cases, yet slightly more favorable for the teflate (about 15 kJ mol^−1^ at the maximum, Table 2).

**Table 2:** Calculated *V*^MnX^_xc_ (kJ mol^−1^) and QTAIM charges (|*e*^−^|) for [MnX_5_]^2−^ complexes (X = OTeF_5_, F)

[MnX_5_]^2−^		*V* ^MnX^_xc_		*q* (Mn)^a^	*q* (X)^a^
Geometry	Position	X = OTeF_5_	X = F		
SPY-5	Basal	−330.3	−315.5	1.90 (1.96)	−0.76 (−0.78)
	Apical	−248.8	−240.4		−0.86 (−0.85)
TBPY-5	Axial	−352.6	−339.5	1.90 (1.97)	−0.74 (−0.76)
	Equatorial	−287.4	−271.1		−0.81 (−0.82)

a Charges correspond to [Mn(OTeF_5_)_5_]^2−^, while values in parentheses are those of [MnF_5_]^2−^.

Finally, the charge of the Mn center is almost the same for the teflate and the fluoride species within each set of compounds, namely the Mn(ii) and the Mn(iii) complex anions, which is in agreement with the similar electronegative character of both groups.^27,28,30^ This way, for the [MnX_4_]^2−^ anions, the charge of the Mn is 1.58 |*e*^−^| when X = OTeF_5_ and 1.54 |*e*^−^| when X = F, similarly to what happens in the Co-based systems (Table 1). In this line, when comparing both Mn(iii) species (Table 2), the Mn has a charge of 1.90 |*e*^−^| in [Mn(OTeF_5_)_5_]^2−^ (for both geometries) and *ca.* 1.96 |*e*^−^| in [MnF_5_]^2−^. All in all, the teflate ligand causes similar effects on the manganese center as the fluoride, therefore allowing a similar stabilization of the oxidation states +II and +III.

## Conclusions

In this work, two unprecedented motifs in the coordination chemistry of manganese with all identical monodentate O-donor ligands are reported and their characterization and properties are investigated by means of theory and experiment. The reaction of [MnCl_4_]^2−^ with AgOTeF_5_ results in the Mn^II^ anion [Mn(OTeF_5_)_4_]^2−^, which displays a distorted tetrahedral structure in the solid state. The nature of the Mn(ii) center was investigated by EPR spectroscopy and magnetic susceptibility measurements, indicating a high spin d^5^ electronic configuration (*S* = 5/2). Additionally, DFT and SA-CASSCF/NEVPT2 calculations show a *pseudo*-^6^A_1_ sextet as the ground state, which is single-reference. On the other hand, when [MnCl_4_]^2−^ or [Mn(OTeF_5_)_4_]^2−^ is reacted with ClOTeF_5_, oxidation of the manganese center takes place to yield the Mn^III^ anion [Mn(OTeF_5_)_5_]^2−^. This species exhibits preferentially a square pyramidal geometry instead of a trigonal bipyramidal one and contains a Mn(iii) center with a high spin d^4^ electronic configuration (*S* = 2), as determined experimentally and backed by theoretical calculations. A bond analysis through the IQA energy decomposition scheme in these [Mn(OTeF_5_)_*n*_]^2−^ anions (*n* = 4, 5) was performed and shows that, in comparison with the hypothetical mononuclear fluoromanganates, the Mn–OTeF_5_ interactions are slightly stronger than the Mn–F ones. Additionally, the charge in the Mn center is virtually the same in both [Mn^II^X_4_]^2−^ analogues (X = OTeF_5_, F), as well as in the [Mn^III^X_5_]^2−^ pair of complexes (X = OTeF_5_, F).

The teflate ligand is known to exhibit similar electronic properties to fluoride, but normally leads to mononuclear species, *i.e.*, it is much less prone to bridge metal centers.^27–30^ In fact, it is this combination of properties that has enabled the formation of the unique compounds reported in this work. In this regard, fluoride is only able to stabilize medium oxidation states of manganese,^15–17^ something also shown now to be possible for the teflate, yet incorporating a novel homoleptic coordination environment of all O-donor monodentate ligands.

## Data availability

Data supporting this manuscript are available in the ESI† and also upon request.

## Author contributions

A. P.-B., J. S. S. and A. N. H. performed synthetic work and collected vibrational spectroscopy data. A. P.-B. interpreted the spectroscopic data. J. M. performed and interpreted theoretical calculations. K. B. K. and C. L. performed and analysed magnetic susceptibility measurements. J. S. and C. T. performed and analysed EPR measurements. K. F. H. and A. W. collected the XRD data and solved and refined the crystal structures. A. P.-B. and S. R. conceptualized and coordinated the project. A. P.-B. and J. M. wrote the manuscript. All authors discussed and commented on the manuscript. A. P.-B., J. M. and S. R. revised the manuscript.

## Conflicts of interest

There are no conflicts to declare.

## Supplementary Material

SC-015-D4SC00543K-s001

SC-015-D4SC00543K-s002
