# Supplementary material for: Questing for homoleptic mononuclear manganese complexes with monodentate O-donor ligands
Source: Chem Sci. 2024 Mar 5;15(15):5564–72. doi: 10.1039/d4sc00543k (PMC11023055; doi:10.1039/d4sc00543k)
Supplement: SC-015-D4SC00543K-s001 [file SC-015-D4SC00543K-s001.pdf]

Electronic Supplementary Material (ESI) for Chemical Science.
This journal is © The Royal Society of Chemistry 2024

Supporting Information

for

Questing for Homoleptic Mononuclear Manganese Complexes with Monodentate O-Donor Ligands

Alberto Pérez-Bitrián,* Julen Munárriz,* Konstantin B. Krause, Johanna Schlögl, Kurt. F. Hoffmann, Johanna S. Sturm, Amiera N. Hadi, Christian Teutloff, Anja Wiesner, Christian Limberg, Sebastian Riedel*

Table of contents

1. Experimental section S2
2. IR spectra S5
3. Crystal data S7
4. Magnetic susceptibility measurements S11
5. EPR spectra S18
6. Quantum-chemical calculations S20
7. Bond Valence Sum analysis S45
8. References S46

1. Experimental section

1.1. General procedures and materials

All experiments were performed under rigorous exclusion of moisture and oxygen using standard Schlenk techniques. Solids were handled in a MBRAUN UNIlab plus glovebox under an argon atmosphere (O_2 < 0.5 ppm, H_2O < 0.5 ppm). Solvents were dried using a MBraun SPS-800 solvent system (CH_2Cl_2, *n*-pentane), or over 3 Å molecular sieves (*o*-difluorobenzene (*o*DFB)). $[NEt_4]_2[MnCl_4]$,[1] $AgOTeF_5$[2, 3] and $ClOTeF_5$[4] were prepared as described elsewhere. $[PPh_4]_2[MnCl_4]$ was prepared following an analogous synthetic procedure to that used for $[NEt_4]_2[MnCl_4]$.[1] All other reagents were purchased from standard commercial suppliers and used as received. Elemental analyses (CHNS) were carried out using a VARIO EL elemental analyzer. IR spectra were measured on neat solid samples at room temperature inside a glovebox under an argon atmosphere using a Bruker ALPHA FTIR spectrometer with a diamond ATR attachment with 32 scans and a resolution of 4 cm^{-1}. EPR spectra of **1** were recorded in the X-band at 298 K with a Magnetech MS 5000 spectrometer. W-band measurements were performed at 20 K with a Bruker Elexsys E680. The samples were sealed in argon atmosphere inside 50 µL Hirschmann glass capillaries. Simulations were performed with Easyspin.[5] Crystal data were collected with MoKα radiation on a Bruker D8 Venture diffractometer with a CMOS area detector. Single crystals were picked at −40 °C under nitrogen atmosphere and mounted on a 0.15 mm Mitegen micromount using perfluoroether oil. The structures were solved with the ShelXT[6] structure solution program using intrinsic phasing and refined with the ShelXL[7] refinement package using least squares minimizations by using OLEX2.[8] The program Diamond V4.6.4 was used for visualization.[9] CCDC 2287496 contains the supplementary crystallographic data for this paper. These data are provided free of charge by The Cambridge Crystallographic Data Centre. Crystal data and other details of the structure analyses are summarized in Table S1 and Table S2. Suitable crystals for X-ray diffraction studies were obtained as indicated in the corresponding experimental entry.

1.2. Synthesis of $[NEt_4]_2[Mn(OTeF_5)_4]$ (**1**)

A mixture of $[NEt_4]_2[MnCl_4]$ (50 mg, 109 µmol, 1 equiv.) and $AgOTeF_5$ (152 mg, 437 µmol, 4 equiv.) in CH_2Cl_2 (10 mL) was stirred in the dark at room temperature for

2 h. The resulting suspension was filtered to separate the colorless solution from the white insoluble solid (AgCl), which was subsequently extracted with more CH_2Cl_2 (2 x 5 mL). The colorless filtrate was evaporated to dryness, rendering an off-white solid that was dried under vacuum and identified as compound **1** (95 mg, 75 µmol, 68%).

IR (ATR, 298 K, Figure S1): $\tilde{\nu}$ / cm^{-1} = 3001 (w), 2959 (w), 2856 (w), 1654 (w), 1486 (m), 1459 (w), 1443 (w), 1396 (w), 1368 (w), 1305 (w), 1241 (w), 1186 (w), 1173 (w), 1070 (w), 1053 (w), 1032 (w), 1001 (w), 854 (m Te−O), 783 (m), 661 (vs, Te−F), 615 (m), 463 (w), 413 (m, Mn−O).

Elemental analysis calcd (%) for $C_{16}H_{40}F_{20}MnN_2O_4Te_4$: C 15.1, H 3.2, N 2.2; found: C 15.6, H 3.8, N 2.2.

1.3. Synthesis of $[PPh_4]_2[Mn(OTeF_5)_4]$ (**1***)

By using the procedure described for the synthesis of **1**, compound **1*** was prepared starting from $[PPh_4]_2[MnCl_4]$ (50 mg, 57 µmol, 1 equiv.) and $AgOTeF_5$ (79 mg, 228 µmol, 4 equiv.). Complex **1*** was obtained as an off-white solid (25 mg, 14.8 µmol, 26%).

IR (ATR, 298 K, Figure S3): $\tilde{\nu}$ / cm^{-1} = 2958 (w), 2924 (w), 2854 (w), 1652 (w), 1586 (w), 1485 (m), 1438 (w), 1398 (w), 1378 (w), 1341 (w), 1316 (w), 1298 (w), 1260 (w), 1186 (w), 1169 (w), 1108 (m), 1074 (w), 1027 (w), 997 (w), 850 (m, sh, Te−O), 760 (w), 724 (m), 691 (m), 667 (vs, Te−F), 616 (m), 524 (vs), 447 (w), 415 (m, Mn−O).

1.4. Crystal growth of $[PPh_4]_2[Mn(OTeF_5)_4]$ (**1***)

A saturated solution of **1*** in 1 mL *o*DFB was placed in a Y-shaped schlenk tube with a greaseless Teflon stopcock. In the side arm, 3 mL *n*-pentane were added. After some days at room temperature, colorless single crystals suitable for X-ray diffraction were obtained.

1.5. Synthesis of $[NEt_4]_2[Mn(OTeF_5)_5]$ (**2**)

Method A: $[NEt_4]_2[MnCl_4]$ (100 mg, 219 µmol, 1 equiv.) was weighed into a Schlenk flask with a greaseless Teflon stopcock. After cooling down to −196 °C, $ClOTeF_5$ (420 mg, 1.53 mmol, 7 equiv.) was condensed onto it. The mixture was allowed to warm

to room temperature under stirring, giving rise to the evolution of Cl_2 as a yellow gas, whereas the solid turned initially red and eventually dark blue. After 1 h of stirring, all volatiles were removed and the product was dried overnight under vacuum, leading to a deep blue solid, which was identified as compound **2** (330 mg, 219 µmol, quant.).

Method B: Compound **1** (50 mg, 39 µmol, 1 equiv.) was weighed into a Schlenk flask with a greaseless Teflon stopcock. After cooling down to −196 °C, $ClOTeF_5$ (22 mg, 79 µmol, 2 equiv.) was condensed onto it. The mixture was allowed to warm to room temperature under stirring, giving rise to a dark blue solid and the evolution of Cl_2 as a yellow gas. After 1 h of stirring, all volatiles were removed and the product was dried overnight under vacuum, leading to a deep blue solid, which was identified as compound **2** (59 mg, 39 µmol, quant.).

IR (ATR, 298 K, Figure S4): $\tilde{\nu}$ / cm^{-1} = 3002 (w), 1486 (m), 1457 (w), 1443 (w), 1417 (w), 1396 (w), 1369 (w), 1188 (w), 1173 (w), 1068 (w), 1053 (w), 1000 (w), 918 (w), 827 (s Te−O), 784 (m), 693 (s, Te−F), 670 (vs, Te−F), 632 (m), 483 (m, Mn−O), 417 (w, Mn−O).

Elemental analysis calcd (%) for $C_{16}H_{40}F_{25}MnN_2O_5Te_5$: C 12.7, H 2.7, N 1.9; found: C 13.2, H 2.6, N 3.2.

1.6. Crystal growth of $[NEt_4]_2[Mn(OTeF_5)_5]$ (**2**)

Blue single crystals to be analyzed by X-ray diffraction were obtained by slow diffusion of a layer of *n*-pentane (1.5 mL) into a solution of compound **2** in *o*DFB (ca. 10 mg in 1 mL) at −24 °C.

2. IR Spectra

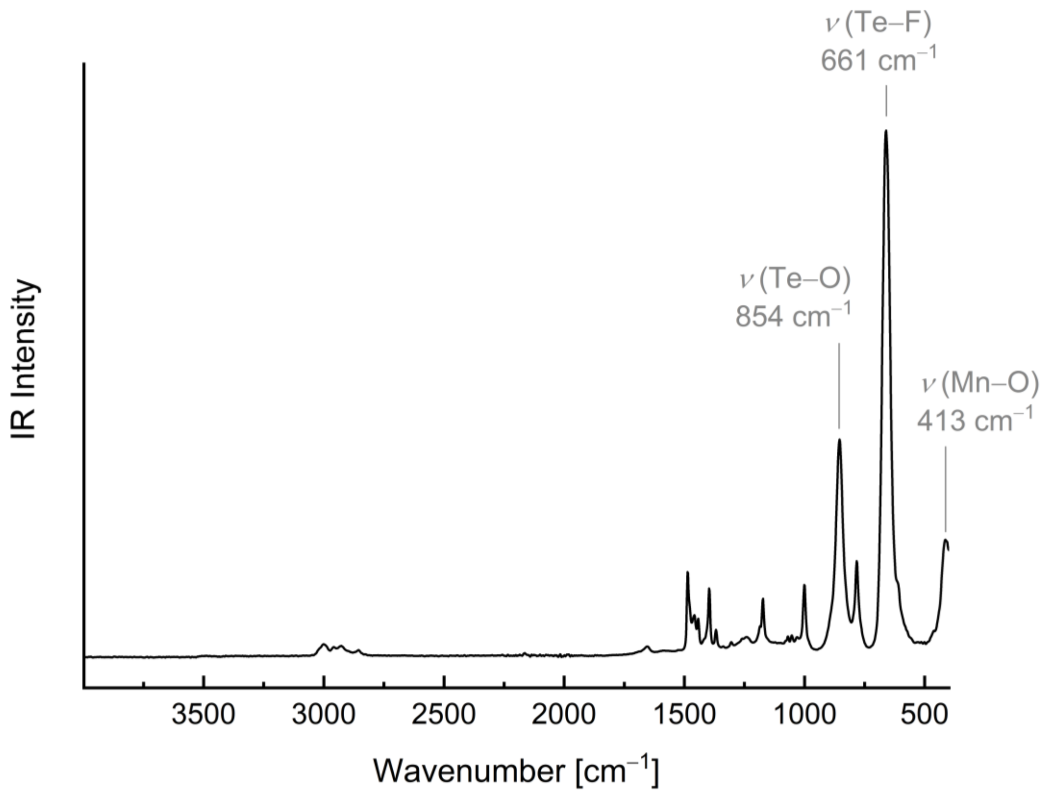

Figure S1. IR spectrum (ATR, 298 K) of $[NEt_4]_2[Mn(OTeF_5)_4]$ (**1**).

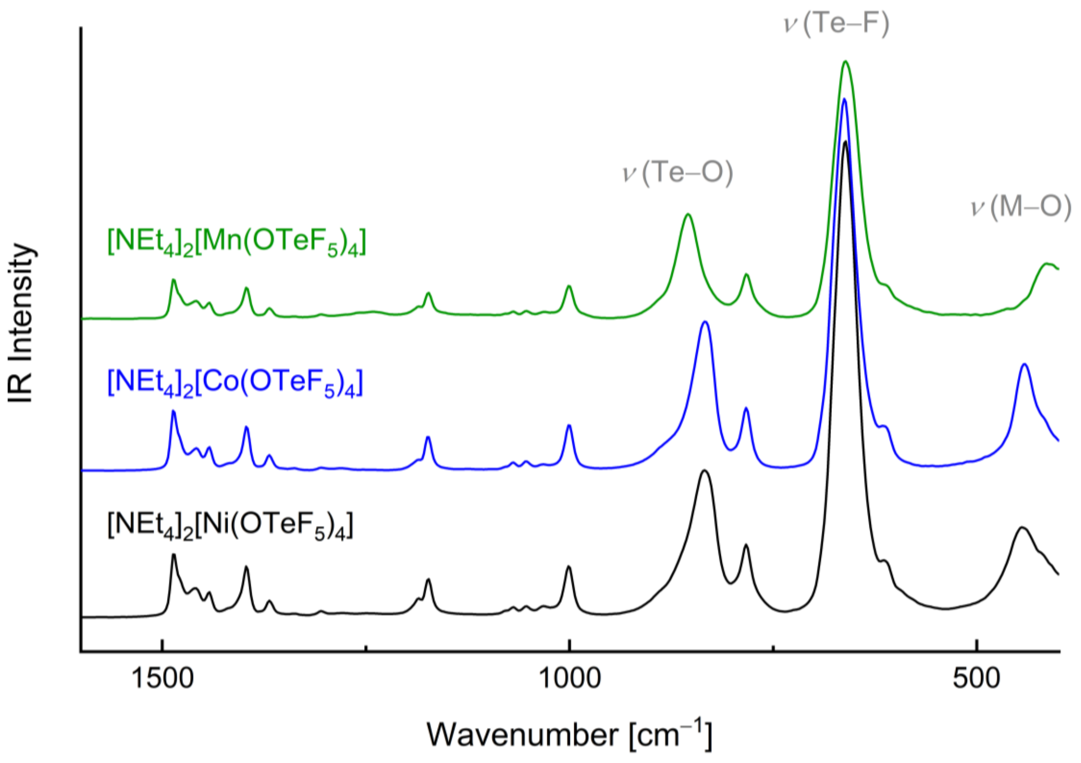

Figure S2. Comparison of the IR spectra (ATR, 298 K) of $[NEt_4]_2[M(OTeF_5)_4]$ salts (M = Ni, Co, Mn).

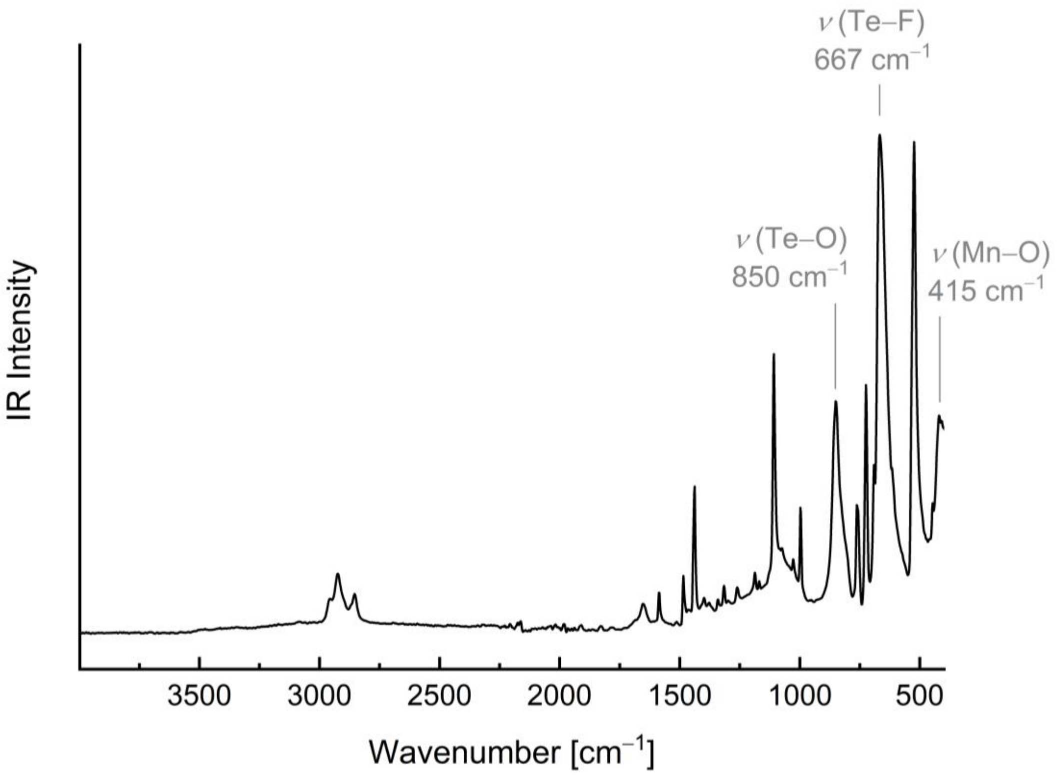

Figure S3. IR spectrum (ATR, 298 K) of $[PPh_4]_2[Mn(OTeF_5)_4]$ (**1***).

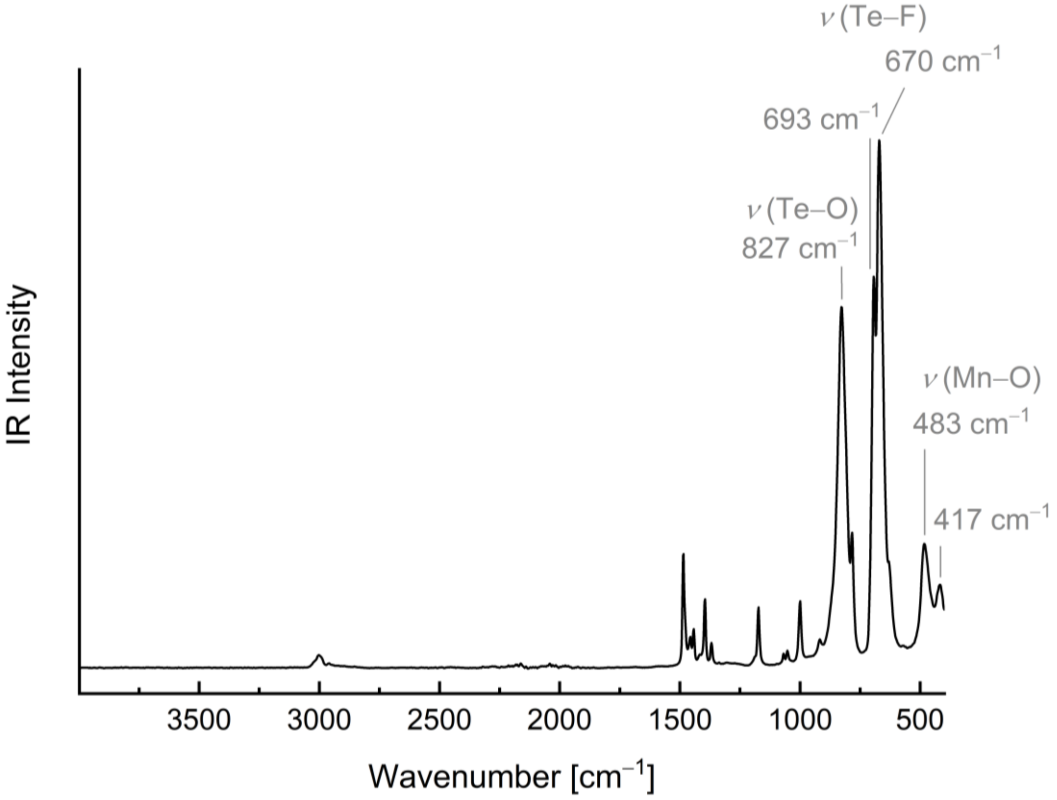

Figure S4. IR spectrum (ATR, 298 K) of $[NEt_4]_2[Mn(OTeF_5)_5]$ (**2**).

3. Crystal data

3.1. Summary of crystal data and structure refinement

Table S1. Crystal data and structure refinement for compound $[PPh_4]_2[Mn(OTeF_5)_4]$ (**1***).

Empirical formula	$C_{48}H_{40}F_{20}MnO_4P_2Te_4$
Formula weight	1688.08
Temperature [K]	273.15
Crystal system	tetragonal
Space group	$I4_1/a$
a [pm]	1186.07(3)
b [pm]	1186.07(3)
c [pm]	3983.39(19)
α [°]	90
β [°]	90
γ [°]	90
Volume [Å^3]	5603.7(4)
Z	4
ρ_{calc} [g·cm^{-3}]	2.001
μ [mm^{-1}]	2.442
F(000)	3212.0
Crystal size [mm^3]	0.174 × 0.13 × 0.087
Radiation	MoKα (λ = 0.71073)
2Θ range for data collection [°]	4.604 to 52.718
Index ranges	$-14 \leq h \leq 14$, $-14 \leq k \leq 14$, $-49 \leq l \leq 49$
Reflections collected	49322
Independent reflections	2771 [R_{int} = 0.0521, R_{sigma} = 0.0214]
Data/restraints/parameters	2771/0/179
Goodness-of-fit on F^2	1.133
Final *R* indexes [$I >= 2\sigma(I)$]	R_1 = 0.0299<br>wR_2 = 0.0644
Final *R* indexes [all data]	R_1 = 0.0393<br>wR_2 = 0.0712
Largest diff. peak/hole [e·Å^{-3}]	0.83/−0.93
CCDC number	2287496

Table S2. Crystal data and structure refinement for compound $[NEt_4]_2[Mn(OTeF_5)_5]$ (**2**).

Empirical formula	$C_{16}H_{40}F_{25}MnN_2O_5Te_5$
Formula weight	1508.44
Temperature [K]	273.15
Crystal system	orthorhombic
Space group	*Pnna*
a [pm]	1757.07(11)
b [pm]	1888.73(9)
c [pm]	1248.39(5)
α [°]	90
β [°]	90
γ [°]	90
Volume [Å^3]	4142.9(4)
Z	4
ρ_{calc} [g·cm^{-3}]	2.418
μ [mm^{-1}]	2.418
F(000)	2.418
Crystal size [mm^3]	0.38 × 0.21 × 0.041
Radiation	MoKα (λ = 0.71073)
2Θ range for data collection [°]	4.002 to 52.814
Index ranges	−13 ≤ h ≤ 21, −23 ≤ k ≤ 23, −15 ≤ l ≤ 14
Reflections collected	14526
Independent reflections	4049 [R_{int} = 0.0519, R_{sigma} = 0.0492]
Data/restraints/parameters	4049/15/237
Goodness-of-fit on F^2	1.285
Final *R* indexes [I >= 2σ (I)]	R_1 = 0.1256<br>wR_2 = 0.2648
Final *R* indexes [all data]	R_1 = 0.1417<br>wR_2 = 0.2731
Largest diff. peak/hole [e·Å^{-3}]	1.90/−2.51

3.2. Molecular structure of $[PPh_4]_2[Mn(OTeF_5)_4]$ (**1***) in the solid state

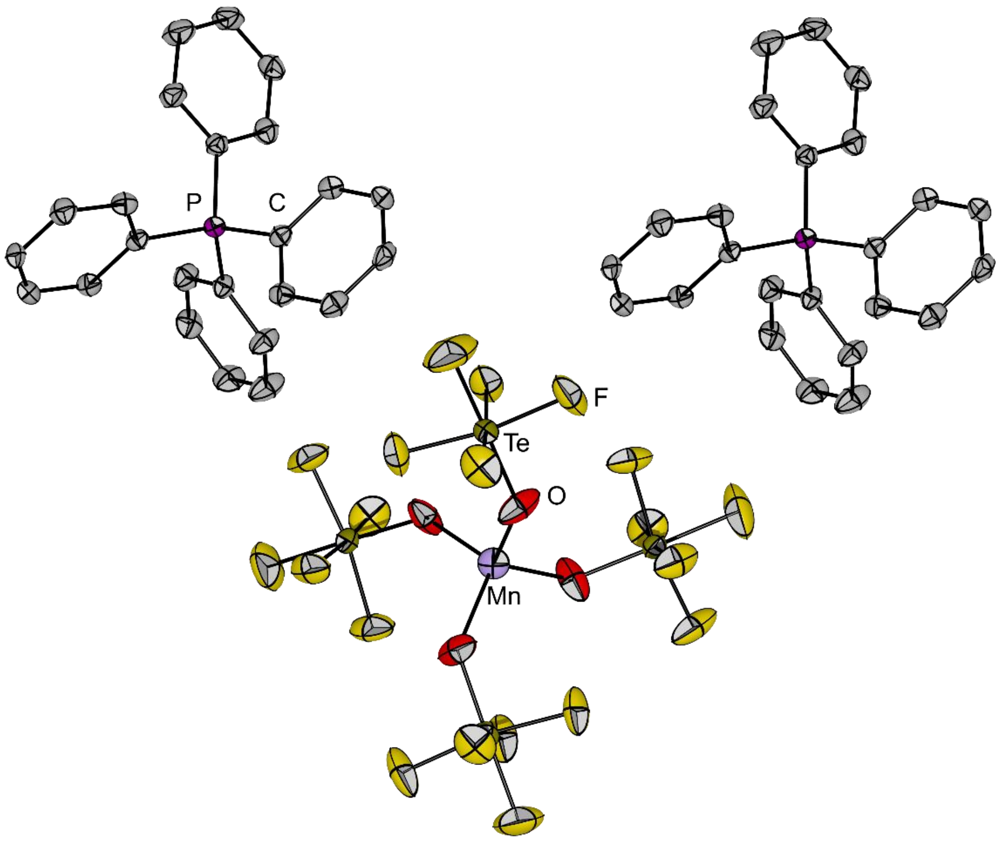

Figure S5. Molecular structure of $[PPh_4]_2[Mn(OTeF_5)_4]$ (**1***) in the solid state. Hydrogen atoms have been omitted for clarity. Displacement ellipsoids set at 50% probability.

3.3. Molecular structure of $[NEt_4]_2[Mn(OTeF_5)_5]$ (**2**) in the solid state

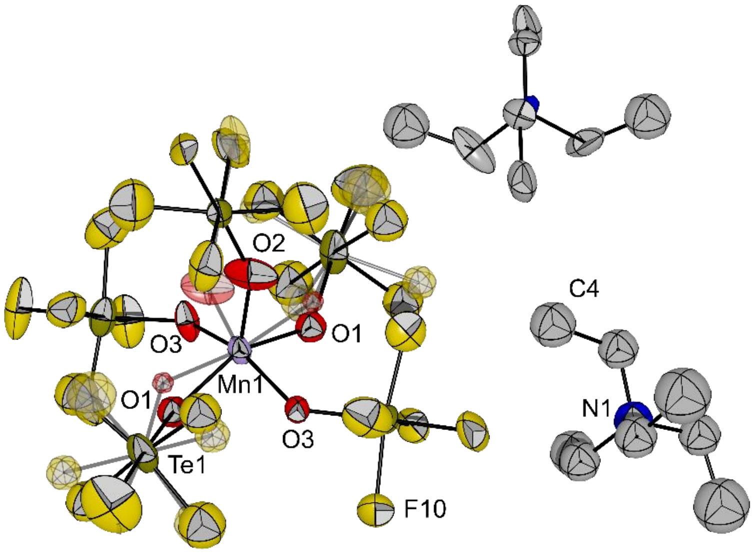

Figure S6. Molecular structure of $[NEt_4]_2[Mn(OTeF_5)_5]$ (**2**) in the solid state. Hydrogen atoms have been omitted for clarity. Displacement ellipsoids set at 50% probability. The two sets of disordered O and F atoms found in the $[OTeF_5]^-$ ligands containing O1 and O2 are shown.

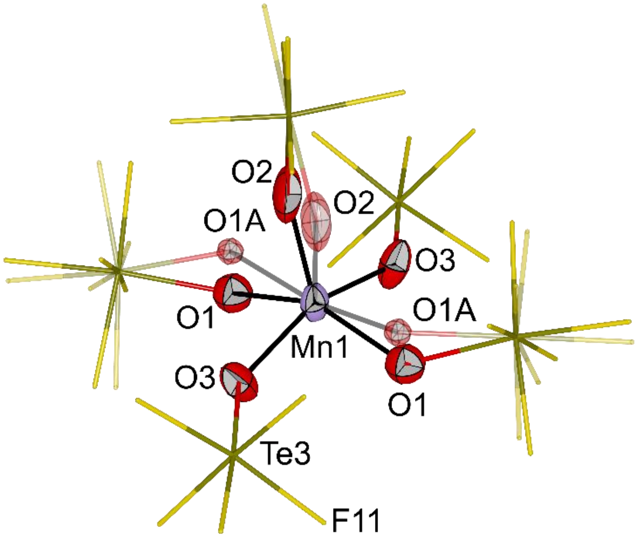

Figure S7. Molecular structure of the $[Mn(OTeF_5)_5]^{2-}$ anion in the solid state as found in crystals of **2**, highlighting the coordination sphere around the Mn(III) center. Displacement ellipsoids set at 50% probability. The two sets of disordered O and F atoms found in the $[OTeF_5]^-$ ligands containing O1 and O2 are shown.

4. Magnetic susceptibility measurements

Magnetic measurements were performed with a QuantumDesign MPMS3 SQUID magnetometer. The samples were prepared in a glovebox in VSM powder capsules which were sealed with a piece of Teflon tape. Both the capsules and the Teflon tape were dried in a Schlenk flask under vacuum at 110 °C for five days. A brass sample holder was used. The measurement was carried out in VSM mode from 2 K to 300 K in a magnetic field of 7 T after cooling down at zero field. A background correction was applied by subtracting the magnetic moments of an empty capsule sealed with a piece of Teflon tape using the same measurement sequence as for the sample. A diamagnetic correction was performed using Pascal's constants.[10] The experimental magnetic data for complex $[NEt_4]_2[Mn(OTeF_5)_4]$ (**1**) and complex $[NEt_4]_2[Mn(OTeF_5)_5]$ (**2**) are collected in Table S3 and Table S4, respectively. The simulation of the experimental magnetic data was performed with N. F. Chilton's PHI software (version 3.1.6)[11] using the following spin Hamiltonian:

$$\hat{H} = g\mu_B B_0 \hat{S} + \hat{S} D \hat{S}$$

The experimental magnetic data, together with their corresponding fits, are shown in Figure 1c and Figure 2, respectively. The fit parameters for compounds **1** and **2** are collected in Table S5).

Table S3. Experimental magnetic data for $[NEt_4]_2[Mn(OTeF_5)_4]$ (**1**) obtained from the SQUID measurement.[a]

T **[K]**	**Magnetic Field [Oe]**	**Magnetic Moment [emu]**	χ_{mol} **[cm³ mol⁻¹]**	χ_p[b] **[cm³ mol⁻¹]**	μ_{eff} **[μB]**
2.000	69999.58	0.24591	0.39131	0.39184	2.5034
2.104	69999.58	0.24564	0.39087	0.39141	2.5662
2.213	69999.58	0.24530	0.39033	0.39087	2.6302
2.328	69999.58	0.24522	0.39021	0.39074	2.6972
2.448	69999.58	0.24475	0.38945	0.38999	2.7635
2.574	69999.58	0.24428	0.38872	0.38925	2.8305
2.709	69999.58	0.24353	0.38752	0.38805	2.8997
2.850	69999.58	0.24288	0.38648	0.38702	2.9699
2.999	69999.58	0.24225	0.38548	0.38602	3.0427

3.152	69999.58	0.24166	0.38454	0.38507	3.1157
3.314	69999.58	0.24069	0.38300	0.38353	3.1882
3.488	69999.58	0.23960	0.38126	0.38180	3.2636
3.671	69999.58	0.23861	0.37969	0.38022	3.3409
3.861	69999.58	0.23744	0.37783	0.37836	3.4181
4.062	69999.58	0.23608	0.37566	0.37619	3.4959
4.273	69999.58	0.23466	0.37341	0.37394	3.5747
4.495	69999.58	0.23313	0.37097	0.37150	3.6543
4.728	69999.58	0.23138	0.36818	0.36872	3.7340
4.974	69999.58	0.22952	0.36523	0.36576	3.8143
5.232	69999.58	0.22749	0.36199	0.36252	3.8948
5.506	69999.58	0.22536	0.35861	0.35914	3.9769
5.793	69999.58	0.22314	0.35507	0.35560	4.0590
6.090	69999.58	0.22052	0.35091	0.35144	4.1372
6.406	69999.58	0.21786	0.34667	0.34720	4.2175
6.739	69999.58	0.21507	0.34223	0.34276	4.2980
7.088	69999.58	0.21195	0.33727	0.33780	4.3760
7.457	69999.58	0.20867	0.33204	0.33257	4.4535
7.843	69999.58	0.20529	0.32667	0.32720	4.5304
8.251	69999.58	0.20166	0.32089	0.32143	4.6054
8.679	69999.58	0.19799	0.31506	0.31559	4.6804
9.125	69999.58	0.19406	0.30880	0.30933	4.7513
9.603	69999.58	0.18989	0.30216	0.30269	4.8216
10.102	69999.58	0.18560	0.29533	0.29586	4.8891
10.627	69999.58	0.18118	0.28830	0.28883	4.9545
11.178	69999.58	0.17651	0.28087	0.28140	5.0157
11.759	69999.58	0.17182	0.27341	0.27395	5.0756
12.369	69999.58	0.16693	0.26563	0.26616	5.1313
13.012	69999.58	0.16208	0.25791	0.25844	5.1859
13.688	69999.58	0.15714	0.25005	0.25058	5.2375
14.398	69999.58	0.15222	0.24222	0.24275	5.2870
15.146	69999.58	0.14728	0.23436	0.23489	5.3341
15.931	69999.58	0.14242	0.22663	0.22716	5.3798
16.759	69999.58	0.13751	0.21881	0.21935	5.4220
17.629	69999.58	0.13255	0.21091	0.21144	5.4599

18.543	69999.58	0.12768	0.20317	0.20370	5.4963
19.506	69999.58	0.12288	0.19553	0.19607	5.5305
20.518	69999.58	0.11814	0.18798	0.18852	5.5619
21.584	69999.58	0.11344	0.18051	0.18104	5.5902
22.704	69999.58	0.10895	0.17337	0.17390	5.6193
23.883	69999.58	0.10444	0.16619	0.16672	5.6432
25.123	69999.58	0.10009	0.15927	0.15980	5.6663
26.427	69999.58	0.09588	0.15257	0.15310	5.6884
27.799	69999.58	0.09176	0.14602	0.14655	5.7081
29.242	69999.58	0.08775	0.13963	0.14016	5.7253
30.760	69999.58	0.08388	0.13348	0.13401	5.7417
32.357	69999.58	0.08012	0.12749	0.12802	5.7559
34.037	69999.58	0.07656	0.12182	0.12235	5.7712
35.804	69999.58	0.07307	0.11627	0.11680	5.7832
37.663	69999.58	0.06971	0.11092	0.11146	5.7941
39.620	69999.58	0.06650	0.10582	0.10635	5.8051
41.674	69999.58	0.06341	0.10090	0.10144	5.8145
43.837	69999.58	0.06044	0.09618	0.09671	5.8229
46.112	69999.58	0.05760	0.09166	0.09219	5.8307
48.505	69999.58	0.05488	0.08732	0.08785	5.8378
51.023	69999.58	0.05229	0.08320	0.08374	5.8455
53.671	69999.58	0.04979	0.07923	0.07977	5.8513
56.456	69999.58	0.04746	0.07552	0.07605	5.8598
59.385	69999.58	0.04518	0.07189	0.07243	5.8650
62.468	69999.58	0.04295	0.06835	0.06888	5.8663
65.711	69999.58	0.04078	0.06489	0.06542	5.8634
69.120	69999.58	0.03873	0.06163	0.06216	5.8619
72.704	69999.58	0.03695	0.05880	0.05933	5.8735
76.477	69999.58	0.03529	0.05615	0.05668	5.8881
80.447	69999.58	0.03359	0.05345	0.05399	5.8936
84.620	69999.58	0.03193	0.05081	0.05135	5.8948
89.009	69999.58	0.03032	0.04825	0.04878	5.8927
93.628	69999.58	0.02886	0.04592	0.04645	5.8979
98.490	69999.58	0.02743	0.04365	0.04418	5.8992
103.600	69999.58	0.02609	0.04152	0.04205	5.9024

108.978	69999.58	0.02480	0.03946	0.03999	5.9036
114.633	69999.58	0.02357	0.03750	0.03803	5.9050
120.583	69999.58	0.02238	0.03561	0.03614	5.9035
126.845	69999.58	0.02127	0.03385	0.03438	5.9056
133.434	69999.58	0.02017	0.03210	0.03264	5.9014
140.360	69999.58	0.01918	0.03051	0.03105	5.9035
147.642	69999.58	0.01823	0.02900	0.02954	5.9056
155.313	69999.58	0.01729	0.02752	0.02805	5.9027
163.376	69999.58	0.01642	0.02613	0.02666	5.9026
171.856	69999.58	0.01562	0.02485	0.02538	5.9064
180.783	69999.58	0.01480	0.02355	0.02408	5.9006
190.171	69999.58	0.01404	0.02235	0.02288	5.8991
200.054	69999.58	0.01332	0.02120	0.02173	5.8970
210.433	69999.58	0.01265	0.02012	0.02065	5.8957
221.362	69999.58	0.01200	0.01909	0.01962	5.8938
232.858	69999.58	0.01137	0.01809	0.01862	5.8890
244.951	69999.58	0.01078	0.01715	0.01768	5.8854
257.667	69999.58	0.01021	0.01624	0.01677	5.8791
271.053	69999.58	0.00966	0.01537	0.01590	5.8714
285.126	69999.58	0.00914	0.01454	0.01508	5.8632
299.969	69999.58	0.00868	0.01381	0.01434	5.8655

[a] m = 11.4 mg [b] χ_p = molar magnetic susceptibility after correction for the diamagnetism of the sample (χ_{dia} = $-531.94\cdot10^{-6}$ cm^3 mol^{-1}, calculated according to Ref. 10).

Table S4. Experimental magnetic data for $[NEt_4]_2[Mn(OTeF_5)_5]$ (**2**) obtained from the SQUID measurement.[a]

T **[K]**	**Magnetic Field [Oe]**	**Magnetic Moment [emu]**	χ_{mol} **[cm^3 mol^{-1}]**	χ_p[b] **[cm^3 mol^{-1}]**	μ_{eff} **[μ$_B$]**
2.000	69999.62	0.15427	0.32274	0.32335	2.2740
2.104	69999.62	0.15415	0.32250	0.32310	2.3319
2.213	69999.62	0.15388	0.32194	0.32254	2.3893
2.328	69999.62	0.15377	0.32170	0.32230	2.4498
2.449	69999.62	0.15364	0.32144	0.32204	2.5113
2.576	69999.62	0.15335	0.32082	0.32142	2.5734

2.710	69999.62	0.15311	0.32031	0.32092	2.6371
2.850	69999.62	0.15279	0.31965	0.32025	2.7019
2.999	69999.62	0.15251	0.31907	0.31967	2.7687
3.154	69999.62	0.15211	0.31823	0.31883	2.8357
3.318	69999.62	0.15181	0.31759	0.31819	2.9056
3.490	69999.62	0.15126	0.31646	0.31706	2.9748
3.671	69999.62	0.15069	0.31526	0.31586	3.0453
3.862	69999.62	0.16073	0.33626	0.33686	3.2256
4.062	69999.62	0.14969	0.31317	0.31377	3.1927
4.273	69999.62	0.14903	0.31180	0.31240	3.2674
4.495	69999.62	0.14830	0.31025	0.31085	3.3429
4.728	69999.62	0.14746	0.30850	0.30910	3.4187
4.974	69999.62	0.14649	0.30647	0.30708	3.4949
5.232	69999.62	0.14557	0.30455	0.30515	3.5733
5.503	69999.62	0.14445	0.30221	0.30282	3.6507
5.789	69999.62	0.14329	0.29979	0.30039	3.7293
6.090	69999.62	0.14185	0.29677	0.29737	3.8059
6.406	69999.62	0.14038	0.29369	0.29429	3.8829
6.738	69999.62	0.13886	0.29052	0.29112	3.9609
7.089	69999.62	0.13719	0.28702	0.28763	4.0380
7.456	69999.62	0.13535	0.28316	0.28376	4.1135
7.809	69999.62	0.13345	0.27919	0.27979	4.1803
8.250	69999.62	0.13115	0.27437	0.27497	4.2595
8.679	69999.62	0.12894	0.26975	0.27035	4.3318
9.129	69999.62	0.12650	0.26466	0.26526	4.4008
9.604	69999.62	0.12395	0.25932	0.25992	4.4681
10.102	69999.62	0.12129	0.25376	0.25436	4.5333
10.627	69999.62	0.11850	0.24792	0.24852	4.5959
11.178	69999.62	0.11555	0.24174	0.24234	4.6546
11.759	69999.62	0.11258	0.23552	0.23612	4.7123
12.369	69999.62	0.10937	0.22881	0.22941	4.7638
13.012	69999.62	0.10626	0.22231	0.22292	4.8164
13.688	69999.62	0.10302	0.21552	0.21612	4.8640
14.398	69999.62	0.09983	0.20885	0.20945	4.9110
15.147	69999.62	0.09657	0.20204	0.20264	4.9546

15.931	69999.62	0.09330	0.19520	0.19580	4.9947
16.759	69999.62	0.09008	0.18845	0.18905	5.0337
17.628	69999.62	0.08679	0.18158	0.18218	5.0680
18.543	69999.62	0.08353	0.17476	0.17536	5.0996
19.506	69999.62	0.08032	0.16804	0.16864	5.1291
20.518	69999.62	0.07716	0.16142	0.16202	5.1562
21.584	69999.62	0.07407	0.15497	0.15557	5.1821
22.705	69999.62	0.07100	0.14854	0.14915	5.2040
23.883	69999.62	0.06802	0.14231	0.14291	5.2247
25.123	69999.62	0.06517	0.13635	0.13695	5.2457
26.427	69999.62	0.06235	0.13044	0.13104	5.2627
27.799	69999.62	0.05964	0.12477	0.12537	5.2795
29.242	69999.62	0.05699	0.11922	0.11982	5.2936
30.760	69999.62	0.05443	0.11387	0.11448	5.3068
32.357	69999.62	0.05199	0.10878	0.10938	5.3203
34.037	69999.62	0.04966	0.10388	0.10449	5.3332
35.804	69999.62	0.04732	0.09899	0.09959	5.3402
37.663	69999.62	0.04517	0.09449	0.09509	5.3520
39.618	69999.62	0.04309	0.09015	0.09075	5.3624
41.674	69999.62	0.04105	0.08588	0.08648	5.3689
43.838	69999.62	0.03912	0.08185	0.08245	5.3766
46.113	69999.62	0.03728	0.07800	0.07860	5.3839
48.506	69999.62	0.03549	0.07425	0.07485	5.3886
51.022	69999.62	0.03377	0.07065	0.07125	5.3919
53.670	69999.62	0.03218	0.06733	0.06793	5.4000
56.459	69999.62	0.03067	0.06416	0.06476	5.4076
59.388	69999.62	0.02921	0.06111	0.06171	5.4139
62.468	69999.62	0.02773	0.05801	0.05861	5.4114
65.712	69999.62	0.02635	0.05514	0.05574	5.4122
69.125	69999.62	0.02502	0.05235	0.05296	5.4107
72.710	69999.62	0.02389	0.04998	0.05058	5.4234
76.486	69999.62	0.02279	0.04768	0.04828	5.4345
80.454	69999.62	0.02170	0.04539	0.04599	5.4400
84.629	69999.62	0.02062	0.04314	0.04374	5.4411
89.021	69999.62	0.01958	0.04097	0.04157	5.4404

93.637	69999.62	0.01863	0.03898	0.03958	5.4443
98.488	69999.62	0.01770	0.03703	0.03763	5.4444
103.600	69999.62	0.01686	0.03527	0.03587	5.4519
108.977	69999.62	0.01645	0.03442	0.03502	5.5245
114.635	69999.62	0.01521	0.03182	0.03242	5.4520
120.600	69999.62	0.01448	0.03028	0.03089	5.4580
126.843	69999.62	0.01375	0.02877	0.02937	5.4587
133.445	69999.62	0.01303	0.02725	0.02785	5.4520
140.361	69999.62	0.01239	0.02592	0.02652	5.4563
147.651	69999.62	0.01209	0.02529	0.02589	5.5291
155.317	69999.62	0.01115	0.02332	0.02392	5.4510
163.373	69999.62	0.01064	0.02225	0.02285	5.4644
171.858	69999.62	0.01013	0.02118	0.02179	5.4720
180.781	69999.62	0.00957	0.02001	0.02061	5.4593
190.187	69999.62	0.00909	0.01901	0.01961	5.4612
200.043	69999.62	0.00863	0.01805	0.01866	5.4632
210.431	69999.62	0.00820	0.01716	0.01776	5.4669
221.364	69999.62	0.00778	0.01627	0.01687	5.4646
232.857	69999.62	0.00737	0.01541	0.01601	5.4610
244.951	69999.62	0.00701	0.01466	0.01526	5.4675
257.673	69999.62	0.00665	0.01392	0.01452	5.4699
271.047	69999.62	0.00632	0.01322	0.01382	5.4735
285.135	69999.62	0.00597	0.01250	0.01310	5.4657
299.932	69999.62	0.00569	0.01190	0.01251	5.4772

[a] m = 10.3 mg [b] χ_p = molar magnetic susceptibility after correction for the diamagnetism of the sample (χ_{dia} = −601.34·10^{-6} cm³ mol^{-1}, calculated according to Ref. 10).

.

Table S5. Fit parameters for compounds $[NEt_4]_2[Mn(OTeF_5)_4]$ (**1**) and $[NEt_4]_2[Mn(OTeF_5)_5]$ (**2**).

		**1**	**2**
**Average *g*-factor**	g_{avg}	**1.995**	**2.223**
	error	0.0003	0.001
**Zero-field splitting**	***D* [cm^{-1}]**	**0.62**	**2.15**
	error	0.03	0.03

5. EPR spectra

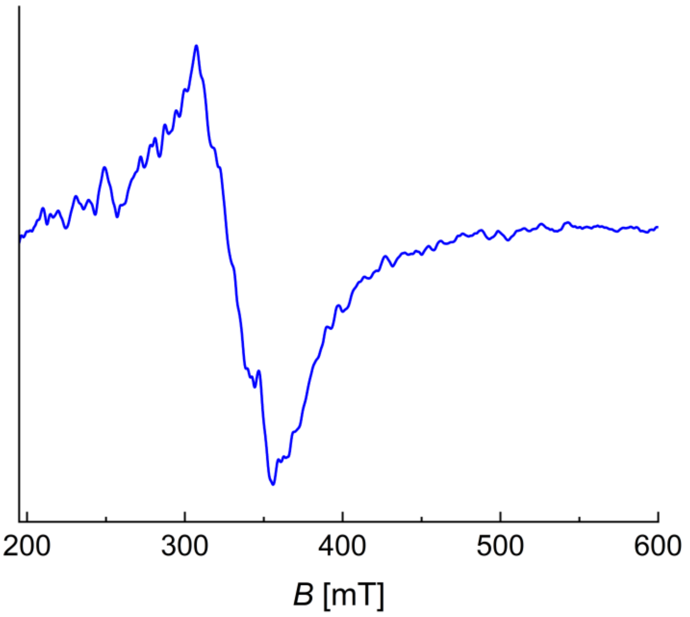

Figure S8. X-band EPR spectrum of $[NEt_4]_2[Mn(OTeF_5)_4]$ (**1**) in CH_2Cl_2 (1.0 mM) at 293 K.

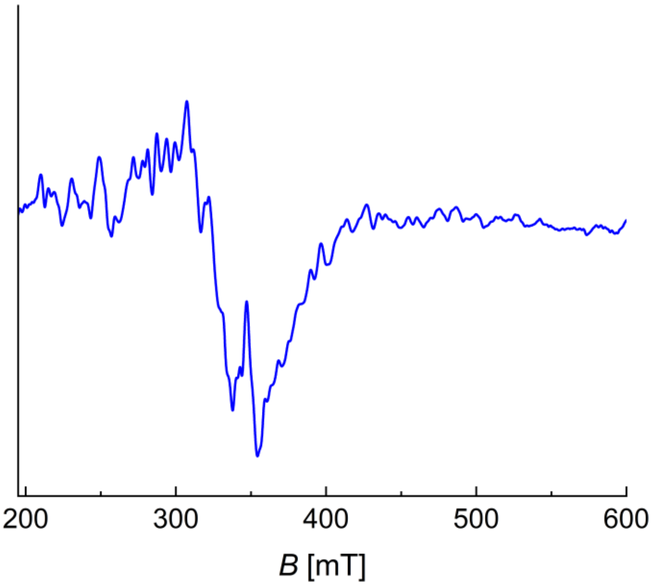

Figure S9. X-band EPR spectrum of $[NEt_4]_2[Mn(OTeF_5)_4]$ (**1**) in CH_2Cl_2 (0.5 mM) at 293 K.

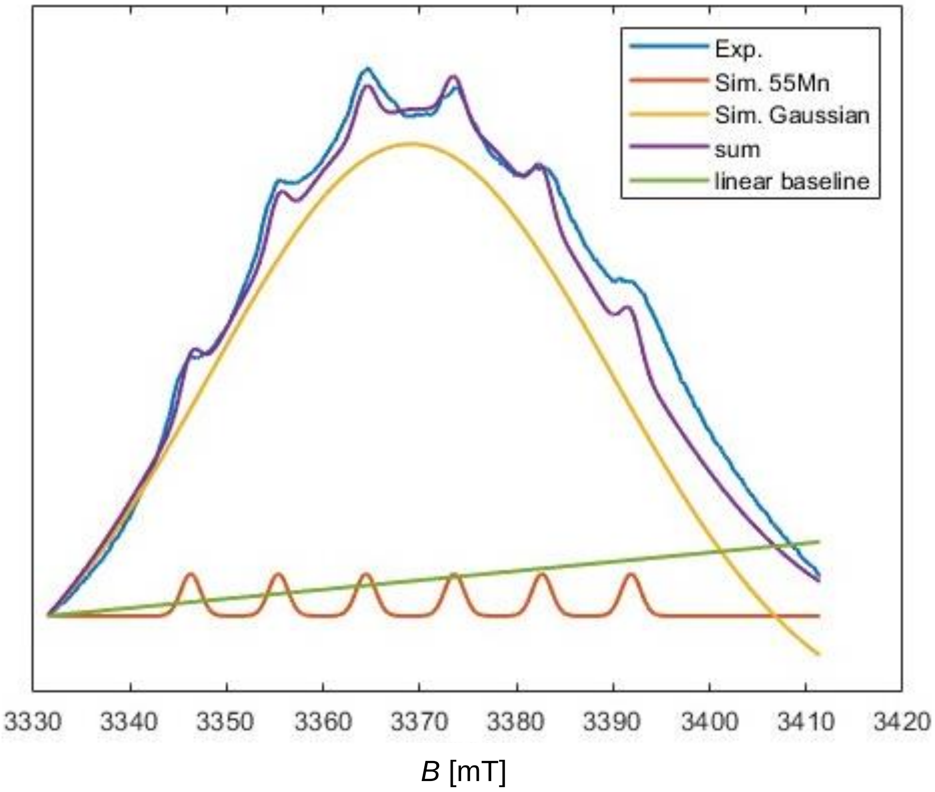

Figure S10. W-band field swept echo EPR spectrum of $[NEt_4]_2[Mn(OTeF_5)_4]$ (**1**) in CH_2Cl_2 (1.0 mM) at 20 K and simulation of the central $m_s = -1/2 \rightarrow m_s = +1/2$ transition, neglecting the zero-field splitting (g_{iso} = 2.00, $A(^{55}Mn)$ = 255 MHz, peak-to-peak linewidth = 2 mT). The substantial broadening of a major part of the spectrum is addressed by a Gaussian line (lw 0 49 mT).

6. Quantum-chemical calculations

6.1. Computational details

Density Functional Theory (DFT) calculations were performed by using the Gaussian 16 program suite.[12] Geometry optimizations were carried out by means of B3LYP[13] (in conjunction with D3BJ dispersion correction scheme[14, 15]), TPSSh,[16-18] M06[19] and M06-L[20] exchange-correlation functionals. In all cases, the triple-zeta def2-TZVP Ahlrichs basis set was employed,[21] in conjunction with an ultrafine integration grid. The nature of stationary points was confirmed by analytical frequency analysis.

Multi-reference calculations on $[Mn(OTeF_5)_4]^{2-}$ and $[Mn(OTeF_5)_5]^{2-}$ structures were performed by means of the ORCA 5.0 package,[22, 23] and considering the B3LYP-optimized geometry. def2-TZVP basis set was used in both CASSCF and NEVPT2 calculations (Refs. 51−54 in the main text), in which we also applied the resolution of identity (RI) approximation to speed up the calculations, in conjunction with def2/JK auxiliary basis set.[24] Strongly contracted NEVPT2 calculations were performed on top of the SA-CASSCF wavefunction to account for dynamic correlation effects. In the active space, we included the Mn−O bonding orbitals based on the 3d orbitals, along with the primarily 3d orbitals of the metal and the corresponding 4d orbitals, to consider the double-d shell correlation effects. This way, for $[Mn(OTeF_5)_4]^{2-}$, the active space is composed by 11 electrons and 13 molecular orbitals CAS(11,13), and is shown in Figure S8. In the state average (SA), we included 1 sextet, 15 quartets and 10 doublet states, which, in a hypothetically perfect tetrahedral geometry, would correspond to the following terms: $^6A_1$, $^4T_1$, $^4T_2$, $^4E(G)$, $^4A_1$, $^4T_2$, $^4E(D)$, $^4A_2$, $^2T_2$, $^2T_1$, $^2A_2$, ^2E and $^2A_1$. The active space for calculations in $[Mn(OTeF_5)_5]^{2-}$ is composed by 12 electrons and 14 molecular orbitals CAS(12,14), and is shown in Figure S9. In the SA we included 5 quintets, 12 triplets and 5 singlets.

Interacting Quantum Atoms (IQA) calculations were performed using the PROMOLDEN code.[25] Molecular orbital representations were made by using VESTA.[26]

6.2. Active space molecular orbitals

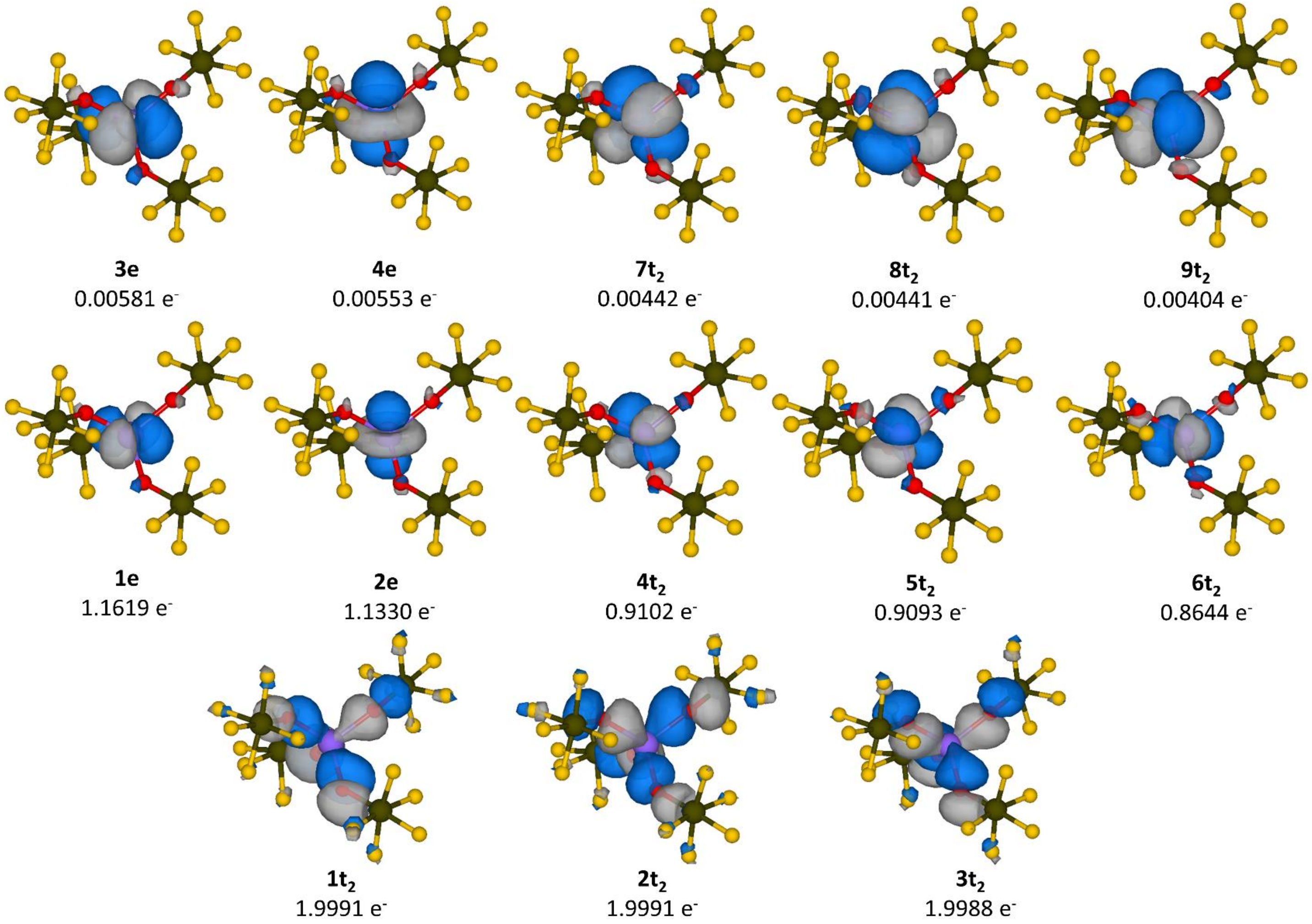

Figure S11. Active space orbitals included in SA-CASSCF(11,13) calculations on $[Mn(OTeF_5)_4]^{2-}$. For the sake of simplicity, the orbital nomenclature is based on a hypothetical tetrahedral structure. The state-average natural population of the various orbitals is also provided. Orbital isosurfaces given at 0.025 au.

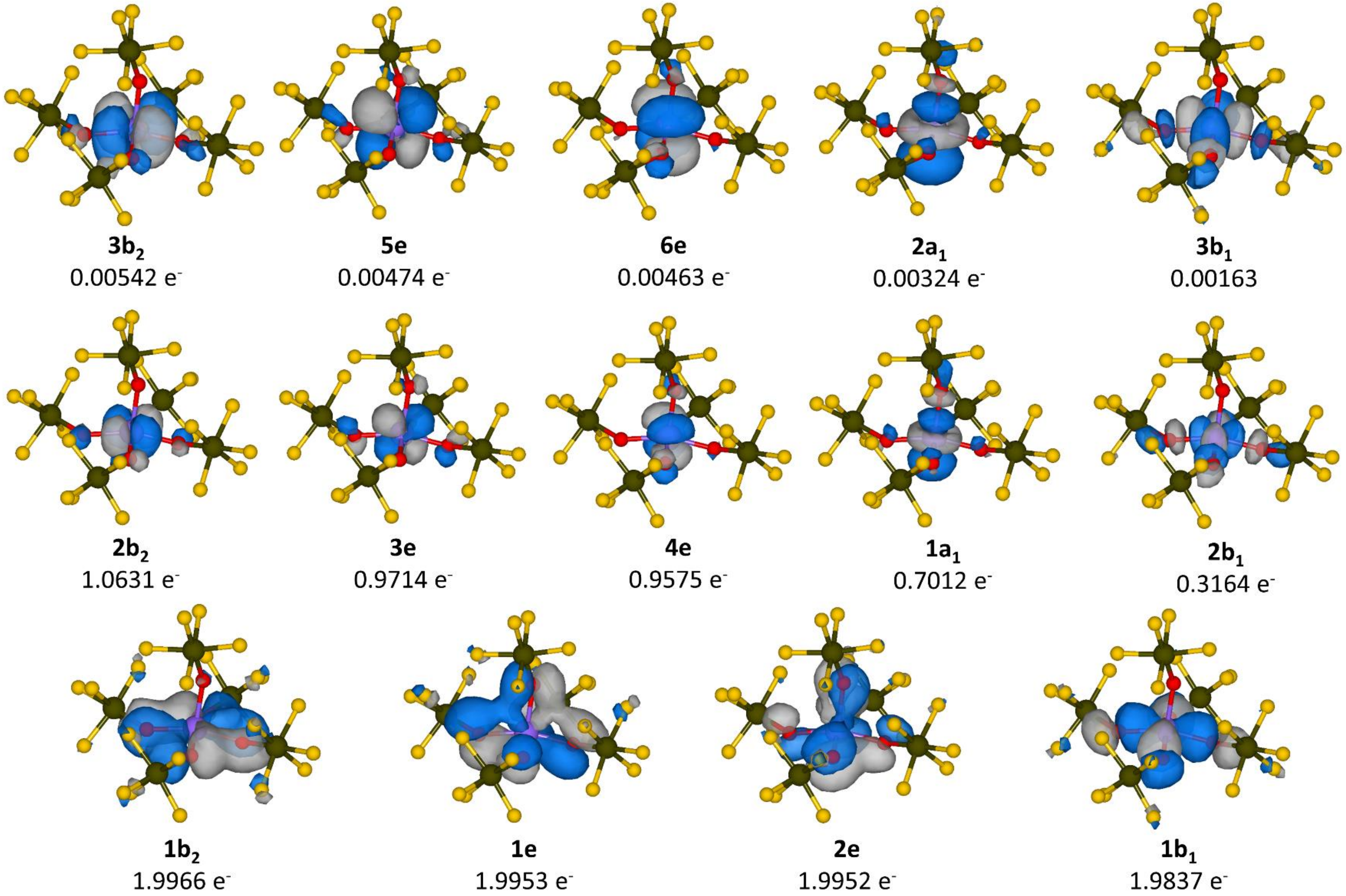

Figure S12. Active space orbitals included in SA-CASSCF(12,14) calculations on $[Mn(OTeF_5)_5]^{2-}$. The orbital nomenclature is based on a hypothetical square-pyramidal structure. The state-average natural population of the various orbitals is also provided. Orbital isosurfaces given at 0.025 au.

6.3. States composition and energy difference

Table S6. SA-CASSCF(11,13)/NEVPT2 states, weights for the most relevant configurations (up to five), and energy difference for the various electronic states computed for $[Mn(OTeF_5)_4]^{2-}$.

State	Root	Mult.	CI-vector		E_{NEVPT2} (au)	ΔE_{NEVPT2}		
			3d[a]	Weight (%)		eV	kJ mol^{-1}	cm^{-1}
0	0	6	11111	98.8	−4515.68340	0.00	0.0	0
1	0	4	21110<br>01112<br>11201<br>11021	90.0<br>6.0<br>1.3<br>1.2	−4515.57440	2.97	286.1	23919
2	1	4	12101<br>21101<br>10121<br>01121<br>11210	56.1<br>26.4<br>5.3<br>3.5<br>3.4	−4515.56847	3.13	301.7	25221
3	2	4	12011<br>21011<br>10211<br>01211<br>11120	57.0<br>25.3<br>5.4<br>3.4<br>3.3	−4515.56839	3.13	301.9	25238
4	3	4	12110<br>11201<br>11021<br>10112<br>11111	34.3<br>21.7<br>21.6<br>13.4<br>7.4	−4515.56400	3.25	313.4	26201
5	4	4	11210<br>11012<br>12101<br>21101<br>10121	24.5<br>20.0<br>16.0<br>13.9<br>7.2	−4515.56326	3.27	315.4	26364
6	5	4	11120<br>11102<br>12011<br>21011<br>10211	24.5<br>20.1<br>15.6<br>14.3<br>7.0	-4515.56325	3.27	315.4	26366
7	6	4	11111	98.6	−4515.56087	3.33	321.7	26889
8	7	4	11111<br>20111<br>02111<br>11201<br>11021	48.9<br>21.4<br>20.2<br>4.1<br>4.0	−4515.56031	3.35	323.1	27012
9	8	4	11111	98.6	−4515.56030	3.35	323.2	27014

10	9	4	10211<br>01121<br>11210<br>01211<br>11120	23.7<br>16.9<br>13.9<br>11.9<br>10.0	−4515.54018	3.90	376.0	31429
11	10	4	10121<br>01211<br>11120<br>01121<br>11210	23.0<br>16.5<br>13.6<br>12.4<br>10.2	−4515.54014	3.90	376.1	31438
12	11	4	12110<br>11201<br>11021<br>10112<br>11111	41.3<br>20.8<br>20.2<br>9.6<br>6.4	−4515.54007	3.90	376.3	31453
13	12	4	11102<br>21011<br>10211<br>10121<br>11120	28.0<br>26.0<br>16.8<br>10.1<br>9.2	−4515.53761	3.97	382.7	31992
14	13	4	11012<br>21101<br>10121<br>10211<br>11210	28.2<br>25.6<br>17.6<br>10.0<br>8.7	−4515.53758	3.97	382.8	32000
15	14	4	01112<br>11021<br>11201<br>21110	74.5<br>11.4<br>10.5<br>2.1	−4515.53388	4.07	392.5	32812
16	0	2	22100<br>20120<br>22010<br>21011<br>02102	61.9<br>6.6<br>6.6<br>5.1<br>3.7	−4515.52103	4.42	426.3	35633
17	1	2	22010<br>20210<br>22100<br>21101<br>02012	61.6<br>6.8<br>6.5<br>5.1<br>3.7	−4515.52096	4.42	426.4	35648
18	2	2	22001<br>12110<br>00221<br>02021<br>02201	62.6<br>13.4<br>3.0<br>2.8<br>2.7	−4515.51728	4.52	436.1	36454

19	3	2	21110<br>01112<br>12110<br>10112<br>20021	74.6<br>9.5<br>4.2<br>1.5<br>1.3	−4515.51509	4.58	441.9	36936
20	4	2	21200<br>21020<br>12002<br>21110<br>11111	23.6<br>22.9<br>10.7<br>9.8<br>8.2	−4515.51566	4.56	440.3	36810
21	5	2	21110<br>11201<br>11021<br>01112<br>11111	48.2<br>13.5<br>13.4<br>6.7<br>4.4	−4515.51231	4.66	449.1	37546
22	6	2	12101<br>21101<br>10121<br>01121<br>21011	49.1<br>22.3<br>10.5<br>5.0<br>3.1	−4515.51048	4.71	453.9	37946
23	7	2	12011<br>21011<br>10211<br>01211<br>21101	49.6<br>21.5<br>10.6<br>4.9<br>3.1	−4515.51042	4.71	454.1	37959
24	8	2	12101<br>11210<br>11012<br>10121<br>21101	36.4<br>13.6<br>11.3<br>9.4<br>8.1	−4515.50890	4.75	458.1	38294
25	9	2	12011<br>11120<br>11102<br>10211<br>21011	36.5<br>13.7<br>11.5<br>9.5<br>7.6	−4515.50885	4.75	458.2	38305

[a] In all the configurations shown, the bonding Mn−O orbitals (MOs $1t_2$−$3t_2$ in Figure S8) were fully occupied, and the 4d orbitals (MOs 3e, 4e and $7t_2$−9 t_2 in Figure S8) were empty. This way, they have been omitted for clarity. The order of the orbital occupancies is the same as in Figure S8.

Table S7. SA-CASSCF(12,14)/NEVPT2 states, weights for the most relevant configurations (up to five), and energy difference for the various electronic states computed for $[Mn(OTeF_5)_5]^{2-}$.

State	Root	Mult.	CI vector[a]			E_{NEVPT2} (au)	ΔE_{NEVPT2}		
			Mn−O	3d	Weight (%)		eV	kJ mol^{-1}	cm^{-1}
0	0	5	2222	11110	96.7	−5356.94199	0.00	0.0	0
			2221	11111	1.0				
			2220	11112	0.4				
1	1	5	2222	11101	98.2	−5356.8644	2.11	203.7	17025
			2221	11102	0.3				
2	0	3	2222	12100	62.7	−5356.85904	2.26	217.8	18203
			2222	11200	14.9				
			2222	21100	7.1				
			2222	10120	5.0				
			2222	02110	2.5				
3	1	3	2222	11200	39.3	−5356.85823	2.28	219.9	18380
			2222	12100	21.4				
			2222	21100	20.2				
			2222	11110	3.5				
			2222	11020	3.3				
4	2	3	2222	21100	42.3	−5356.85603	2.34	225.6	18863
			2222	11200	28.8				
			2222	11110	6.6				
			2222	10210	4.8				
			2222	01120	4.6				
5	2	5	2222	01111	76.6	−5356.85498	2.37	228.4	19093
			2222	11011	20.3				
			1222	11111	0.6				
			2212	11111	0.3				
6	3	5	2222	11011	65.2	−5356.85305	2.42	233.5	19518
			2222	01111	18.5				
			2222	10111	12.9				
			2212	11111	0.9				
7	4	5	2222	10111	83.5	−5356.85295	2.42	233.7	19539
			2222	11011	10.9				
			2222	01111	2.0				
			2122	11111	1.2				
8	3	3	2222	11110	91.8	−5356.84787	2.56	247.1	20655
			2222	21010	1.6				
			2222	20011	1.1				
			2222	01210	0.7				
			2222	11101	0.6				
9	4	3	2222	21010	75.5	−5356.84206	2.72	262.3	21928
			2222	01210	6.0				

			2222	11110	3.4				
			2222	11200	2.9				
			2222	12100	2.9				
10	5	3	2222	20110	75.1	−5356.84064	2.76	266.0	22240
			2222	02110	7.7				
			2222	11110	2.9				
			2222	11020	2.5				
			2222	11200	2.2				
11	6	3	2222	11110	32.1	−5356.83798	2.83	273.0	22824
			2222	12010	21.1				
			2222	21100	19.3				
			2222	10210	15.0				
			2222	12001	3.9				
12	7	3	2222	11110	47.2	−5356.83672	2.86	276.3	23100
			2222	12010	22.3				
			2222	10210	20.1				
			2222	11101	3.6				
			2222	01111	1.0				
13	8	3	2222	11110	88.0	−5356.82831	3.09	298.4	24946
			2222	20011	3.7				
			2222	21010	1.0				
			2222	11101	0.8				
			2222	01210	0.7				
14	0	1	2222	12100	25.6	−5356.8149	3.46	333.6	27888
			2222	11200	25.0				
			2222	02110	10.2				
			2222	22000	9.7				
			2222	20110	7.7				
15	1	1	2222	11110	18.5	−5356.81381	3.49	336.5	28128
			2222	12100	16.3				
			2222	02200	11.7				
			2222	20200	10.7				
			2222	21100	10.3				
16	9	3	2222	11020	54.3	−5356.81389	3.49	336.3	28110
			2222	02110	12.7				
			2222	11011	8.3				
			2222	10120	6.8				
			2222	01210	2.6				
17	2	1	2222	11200	19.7	−5356.81337	3.50	337.7	28225
			2222	11110	15.1				
			2222	22000	14.6				
			2222	02200	10.5				
			2222	21100	5.6				
18	3	1	2222	21100	35.4	−5356.81298	3.51	338.7	28309

			2222	20200	19.2				
			2222	10210	13.1				
			2222	22000	10.7				
			2222	11200	7.3				
			2222	11110	28.9				
			2222	12100	17.4				
19	4	1	2222	21010	8.4	−5356.81158	3.55	342.3	28616
			2222	01210	7.4				
			2222	22000	7.4				
			2222	10120	54.2				
			2222	01210	10.6				
20	10	3	2222	10111	9.0	−5356.81033	3.58	345.6	28893
			2222	11020	5.3				
			2222	02110	3.1				
			2222	10210	32.1				
			2222	12010	24.9				
21	11	3	2222	11101	11.7	−5356.80851	3.63	350.4	29292
			2222	10201	7.5				
			2222	12001	5.7				

[a] Mn−O orbitals refer to bonding MOs $1b_2$, 1e, 2e and $1b_1$, and 3d refer to antibonding orbitals that mainly correspond to 3d orbitals of Mn: $2b_2$, 3e, 4e, $1a_1$ and $2b_1$. The 4d orbitals ($3b_2$, 5e, 6e, $2a_1$ and $3b_1$) were empty in all the vectors shown and have been omitted. The order of the orbital occupancies is the same as in Figure S9.

6.4. DFT-optimized structures

The coordinates of the DFT-optimized structures are provided below. Note that, for $[Mn(OTeF_5)_4]^{2-}$, we only calculated the HS structure, while for $[Mn(OTeF_5)_5]^{2-}$ we considered HS, IS and LS possibilities.

As introduced in the main text, initial geometries of the square pyramid (*SPY*-5) and trigonal bipyramid (*TBPY*-5) geometries converged to slightly different square pyramid structures, which are referred to as *SPY*-5 and *SPY*-5’. In this regard, we note that, in the case of TPSSh, the *SPY*-5’ geometry is closer to a trigonal bipyramid, but we have kept the nomenclature for simplicity. The structure and energy of both structures are very similar (see Table S9). In order to obtain the *TBPY*-5 geometry, we performed a constrained geometry optimization, in which we fixed the angle of O−Mn−O bonds in the equatorial positions at 120° and imposed the Mn and the three O atoms in the equatorial positions to be on the same plane (dihedral angle of 0°). When the electronic

energy is considered (Tables S8 and S9), the square pyramid structure is the most stable for all functionals. Nonetheless, the energy difference is very small, being 8.9 kJ mol^{-1} at the maximum (for M06-L, Table S9). When we compare Gibbs energies (Tables S10 and S11), the square pyramid structure is the most favorable geometry for B3LYP, M06-L and TPSSh functionals, the trigonal bipyramid being 2.7 kJ mol^{-1} higher in energy for M06. In this respect, we note that the energy differences between both are still very small and that small imaginary frequencies (of the order of 20i cm^{-1}) appear when B3LYP and TPSSh functionals are considered (due to the optimization under geometry constraints). This way, we consider the electronic energy to be more reliable to compare the relative stability of both geometries.

Given that IS and LS structures are significantly above in energy, we only provide the most stable structure. We note that there are significant variations depending on the functional. However, as LS and IS structures are very high in energy in all cases when compared to the HS analogues, such differences are not further analyzed. For the sake of completeness, we provide the energy (electronic and Gibbs energy) data of the various considered structures with each functional in Tables S8-S11.

Table S8. Electronic energies (in au) for the various structures considered for $[Mn(OTeF_5)_5]^{2-}$ as a function of the DFT functional.

	HS			IS	LS
	SPY-5	*SPY*-5'	*TBPY*-5		
B3LYP	−5365.63405	−5365.63392	−5365.63161	−5365.59108	−5365.57871
M06-L	−5365.58030	−5365.57868	−5365.57692	−5365.52846	−5365.50181
M06	−5364.48206	−5364.48130	−5364.48052	−5364.41027	−5364.39374
TPSSh	−5364.62045	−5364.61977	−5364.61874	−5364.58440	−5364.57803

Table S9. Electronic energy difference (in kJ mol^{-1}) for the various structures considered for $[Mn(OTeF_5)_5]^{2-}$ as a function of the DFT functional.

	HS			IS	LS
	SPY-5	*SPY*-5'	*TBPY*-5		
B3LYP	0.0	0.4	6.4	112.8	145.3
M06-L	0.0	4.2	8.9	136.1	206.1
M06	0.0	2.0	4.0	188.5	231.9
TPSSh	0.0	1.8	4.5	94.7	111.4

Table S10. Gibbs energies (in au) for the various structures considered for $[Mn(OTeF_5)_5]^{2-}$ as a function of the DFT functional.

	HS			IS	LS
	SPY-5	*SPY*-5'	*TBPY*-5		
B3LYP	−5365.64731	−5365.64733	−5365.64304	−5365.59907	−5365.58881
M06-L	−5365.58874	−5365.58547	−5365.58667	−5365.53612	−5365.50936
M06	−5364.47815	−5364.47802	−5364.47919	−5364.40707	−5364.39170
TPSSh	−5364.63557	−5364.63468	−5364.63131	−5364.59737	−5364.59062

Table S11. Gibbs energy difference (in kJ mol^{-1}) for the various structures considered for $[Mn(OTeF_5)_5]^{2-}$ as a function of the DFT functional.

	HS			IS	LS
	SPY-5	*SPY*-5'	*TBPY*-5		
B3LYP	0.0	0.0	11.3	126.7	153.6
M06-L	0.0	8.6	5.4	138.1	208.4
M06	2.7	3.1	0.0	189.3	229.7
TPSSh	0.0	2.3	11.2	100.3	118.0

With respect to fluoride-based structures ($[MnF_n]^{2-}$ (n = 4, 5), we only considered the HS analogues (the structures are provided below). For $[MnF_4]^{2-}$, we found a tetrahedral geometry, while for $[MnF_5]^{2-}$ we considered both *SPY*-5 and *TBPY*-5 alternatives (see Tables S12 and S13 for absolute and relative electronic and Gibbs energies). The *TBPY*-5 structures do not show any imaginary frequency for any functional, while the *SPY*-5 ones show a single imaginary frequency for B3LYP (9i cm^{-1}) and M06 (32i cm^{-1}), which corresponds to the interconversion to the *TBPY*-5 geometry. With respect to relative electronic energies, the *TBPY*-5 is the global minimum for B3LYP, M06 and M06-L, while it is 0.4 kJ mol^{-1} higher than the *SPY*-5 for TPSSh. When Gibbs energies are considered, the *TBPY*-5 structure is the global minimum in all the cases. However, the energy differences are always very small (being lower than the DFT accuracy) and, as a result, we cannot make a definitive comparison.

Table S12. Electronic (E) and Gibbs (G) energies (in au) for the various structures considered for $[MnF_5]^{2-}$ as a function of the DFT functional.

	SPY-5		*TBPY*-5	
	E	*G*	*E*	*G*
B3LYP	−1650.67392	−1650.69877	−1650.67444	−1650.70131
M06-L	−1650.51730	−1650.54451	−1650.51777	−1650.54478
M06	−1650.41444	−1650.43889	−1650.41530	−1650.44168
TPSSh	−1650.62257	−1650.64922	−1650.62242	−1650.64974

Table S13. Electronic (*E*) and Gibbs (*G*) energy difference (in kJ mol^{-1}) with respect to the global minimum (which takes the value of 0.0 kJ mol^{-1}) for the various structures considered for $[MnF_5]^{2-}$ as a function of the DFT functional.

	Δ*E*		Δ*G*	
	SPY-5	*TBPY*-5	*SPY*-5	*TBPY*-5
B3LYP	1.4	0.0	6.7	0.0
M06-L	1.2	0.0	0.7	0.0
M06	2.3	0.0	7.3	0.0
TPSSh	0.0	0.4	1.3	0.0

$[Mn(OTeF_5)_4]^{2-}$

B3LYP

Te	-2.47316800	-2.13239800	-1.53489900
Te	2.44611900	2.13308000	-1.55376000
Mn	0.00042200	-0.00450800	0.00180100
F	1.98595900	-2.27426200	3.40547800
F	-1.93609000	2.28018400	3.42080800
F	-2.83276100	-2.60946800	0.24750500
F	-0.98856600	-3.28721100	-1.54744400
F	3.29821400	-0.95611900	1.51963900
F	0.95372300	3.27753900	-1.54339100
F	-3.27012700	0.95522800	1.55504400
F	-4.13870200	-1.26144800	-1.62722600
O	-1.53957100	-0.63328600	-1.14971700
O	0.64211200	-1.53192600	1.16567000
O	1.52765000	0.62407600	-1.16927700
O	-0.61904200	1.53106500	1.16735300
F	-3.46244700	-3.68192500	-1.96624500
F	-2.31120000	-1.93678700	-3.40298900
F	4.11687300	1.27475400	-1.66969300
F	2.82178900	2.60103200	0.22759500
F	3.42009500	3.69222600	-1.98546400
F	2.26626300	1.94874900	-3.42146400
Te	2.15523100	-2.44943000	1.53596500
Te	-2.12794500	2.44947100	1.55285100
F	3.71864300	-3.42313200	1.95239400
F	2.60862500	-2.82077200	-0.25017700
F	1.29931300	-4.12138500	1.65481300
F	-1.27167500	4.12212000	1.65604100
F	-3.68690200	3.42348100	1.98512800
F	-2.60347600	2.81448600	-0.22872900

M06

Te	2.28005100	2.31375200	1.43096800
Te	-2.28087800	-2.31388000	1.43035700
Mn	-0.00000900	-0.00016900	0.00023700
F	2.28534100	-2.14249100	-3.29322500
F	-2.28238300	2.14415000	-3.29374800
F	2.71131100	2.52351400	-0.36852300
F	3.30423200	0.75767200	1.49840400
F	0.75837000	-3.30395500	-1.50123300
F	-3.30443700	-0.75745600	1.49936500
F	-0.75758800	3.30433900	-1.49907000
F	1.55674700	4.03224800	1.44964500
O	0.71146300	1.48999400	1.15704100
O	1.49078900	-0.71158500	-1.15586600
O	-0.71205000	-1.49056500	1.15643000
O	-1.49003400	0.71163600	-1.15663500
F	3.90411500	3.18883800	1.74614700
F	2.14466200	2.28205700	3.29388400
F	-1.55826500	-4.03268000	1.44753600
F	-2.71297200	-2.52216200	-0.36910200
F	-3.90515400	-3.18854500	1.74562500

F	-2.14471300	-2.28354700	3.29323600
Te	2.31451300	-2.28010000	-1.43043400
Te	-2.31368600	2.28024600	-1.43087900
F	3.18970900	-3.90394700	-1.74643900
F	2.52174000	-2.71359200	0.36880800
F	4.03318800	-1.55714700	-1.44591500
F	-4.03226100	1.55711900	-1.44895500
F	-3.18872400	3.90424700	-1.74652600
F	-2.52304900	2.71216400	0.36849800

M06-L

Te	2.48117700	2.16388100	1.37931700
Te	-2.48111400	-2.16361400	1.37965400
Mn	0.00004600	0.00002000	0.00004000
F	2.13831200	-2.39486200	-3.26072900
F	-2.13840800	2.39422800	-3.26111600
F	2.88145100	2.35242000	-0.44561400
F	3.42882800	0.54154600	1.42301200
F	0.54142300	-3.42881200	-1.42292100
F	-3.42873200	-0.54126700	1.42338600
F	-0.54155800	3.42864400	-1.42356100
F	1.85436800	3.93545600	1.41949200
O	0.85056000	1.42526000	1.14405200
O	1.42523300	-0.85055700	-1.14401300
O	-0.85047300	-1.42508300	1.14423900
O	-1.42520000	0.85037600	-1.14411700
F	4.17228700	2.95335200	1.65194900
F	2.39494300	2.13837600	3.26075500
F	-1.85435600	-3.93521200	1.41976400
F	-2.88157800	-2.35218200	-0.44522800
F	-4.17221000	-2.95305000	1.65247900
F	-2.39468500	-2.13806800	3.26108400
Te	2.16377400	-2.48120200	-1.37929400
Te	-2.16384500	2.48093700	-1.37969800
F	2.95317700	-4.17233000	-1.65202400
F	2.35231800	-2.88150600	0.44563200
F	3.93538700	-1.85448300	-1.41931400
F	-3.93542000	1.85410600	-1.41956800
F	-2.95335400	4.17196300	-1.65276100
F	-2.35238000	2.88161700	0.44514400

TPSSh

Te	-1.50350800	-2.67260800	2.04344100
Te	1.50348200	2.66959300	2.04698000
Mn	-0.00015000	-0.00005100	-0.00005200
F	-2.41176200	0.69307000	-3.72020600
F	2.41133900	-0.68817700	-3.72116900
F	-2.53411000	-3.15642600	0.54962100
F	-2.74290300	-1.32105800	2.45296800
F	-1.31901300	2.74401100	-2.45269400
F	2.74269700	1.31729200	2.45456400
F	1.31946300	-2.74099400	-2.45596300
F	-0.48054400	-4.23305000	1.81923100
O	-0.34795900	-1.61168900	1.14581700
O	-1.61105700	0.34864700	-1.14666100
O	0.34776600	1.61003400	1.14796100
O	1.61079300	-0.34704000	-1.14712800
F	-2.68136800	-3.79720300	2.99502000
F	-0.69079500	-2.41360800	3.71906400
F	0.48066900	4.23046300	1.82506100
F	2.53395100	3.15534800	0.55370500
F	2.68163600	3.79271500	2.99993400
F	0.69089200	2.40841100	3.72233000
Te	-2.67119400	1.50495000	-2.04424100
Te	2.67123200	-1.50192100	-2.04617900
F	-3.79504900	2.68375900	-2.99552200
F	-3.15523400	2.53489900	-0.55005000
F	-4.23202400	0.48229200	-1.82133500
F	4.23171600	-0.47897000	-1.82222400
F	3.79542500	-2.67920400	-2.99894600
F	3.15576800	-2.53346100	-0.55324600

$[MnF_4]^{2-}$

B3LYP

Mn	0.00044600	-0.00032400	0.00020300
F	1.62346900	-0.84893600	0.89227700
F	-0.92249400	-1.37770700	-1.18275600
F	-1.30362600	0.64690300	1.42465700
F	0.60141300	1.58063900	-1.13474400

$[Mn(OTeF_5)_5]^{2-}$

B3LYP

HS − *SPY*-5

Te	-1.07295700	3.30951500	-0.57826300
Te	0.79925900	-3.36085000	-0.54307800
Mn	-0.12826500	-0.00986200	-0.59297000
F	3.08051400	-0.47100900	-2.51395000
F	-3.39152800	0.34695800	0.70904300
F	-3.82433300	0.30541400	-1.91477000
F	-0.45447000	-3.27481000	0.83392900
F	0.26082300	3.19165600	0.71766400
F	-0.55600300	-3.63838600	-1.80451500
F	0.20231700	3.70029500	-1.89339800
F	-2.41600100	3.71649000	-1.82375800
F	-2.38067700	3.23548600	0.75380300
F	2.18992900	-3.41196000	0.69978100
O	-1.66991400	-1.08115800	-0.95470600
O	1.41539600	1.05838100	-0.94874000
O	-1.18677900	1.52838200	-0.98475000
O	0.94900800	-1.55881000	-0.81795000
F	2.05241100	-3.72716700	-1.88880300
F	-1.04963400	5.16080900	-0.23673900
F	0.72960700	-5.22755700	-0.30464900
O	-0.33229900	0.06063000	1.40950500
Te	-3.46015400	-0.97813200	-0.59914600
Te	3.21281300	0.85864400	-1.19990700
Te	0.52253200	0.17260300	2.99804700
F	-5.32080200	-0.96976700	-0.30607000
F	-3.83004100	-2.31443100	-1.86312600
F	-3.41256900	-2.29380200	0.72785000
F	3.22916000	2.13707200	-2.57351700
F	3.64744300	2.21787600	0.00517000
F	3.50139900	-0.42469800	0.12145200
F	5.06884800	0.74571400	-1.50231700
F	2.02220800	1.10212000	2.36037500
F	-0.27315400	1.80060900	3.49500300
F	1.37827900	0.28330800	4.67784200
F	1.45538900	-1.44653500	2.82089700
F	-0.82847500	-0.73317600	3.94274600

HS − *SPY*-5’

Te	-3.36695700	-0.66054700	-0.29818600
Te	3.52030900	0.41627200	-0.52559400
Mn	0.06340100	0.04478900	-0.39656700
F	-0.00139800	2.19020900	-2.79727500
F	-0.90414100	-3.24238900	0.35917700
F	-0.81745500	-3.30884200	-2.30335500
F	3.30576700	-1.05635300	0.59856600
F	-3.44298500	1.08826800	0.34635700
F	3.52974900	-0.69425700	-2.03379600
F	-4.05963500	-0.07615100	-1.93966800
F	-3.60637500	-2.42322200	-0.88005400
F	-2.95577800	-1.27846000	1.41213800
F	3.83158000	1.50133500	0.96522200
O	0.85263800	-1.57978400	-1.01385700
O	-0.64988700	1.80976900	-0.16862600
O	-1.64903400	-0.61014400	-0.92276400
O	1.75969700	0.88674000	-0.64585100
F	4.03090100	1.84634100	-1.62663000
F	-5.16737900	-0.77333700	0.24361800
F	5.36275100	0.03500700	-0.43667400
O	0.09828900	-0.42559900	1.56435900
Te	0.44631200	-3.35911300	-0.92210800

Te	-0.70348000	3.18711900	-1.36541600
Te	0.09656000	0.40581700	3.17325400
F	0.11124200	-5.21173100	-0.89994000
F	1.76064500	-3.77224700	-2.19381800
F	1.68712600	-3.72268800	0.42781600
F	-2.42920200	2.78976900	-1.97541100
F	-1.43999200	4.37170400	-0.11979300
F	0.99518400	3.85676100	-0.96691700
F	-0.79140200	4.61863900	-2.58433800
F	0.92861200	1.98502500	2.59412200
F	-1.58490200	1.21503000	2.97612000
F	0.09975100	1.22803900	4.87343700
F	1.77364300	-0.26822800	3.69157600
F	-0.71645300	-1.03422900	4.06803800

Te	-2.85237700	1.85717800	-0.30857800
Te	0.03683300	0.08058100	3.28877200
F	4.17222400	-3.24602600	0.12584600
F	4.27143100	-1.12714400	-1.28000600
F	3.48361400	-1.05880800	1.22677600
F	-4.13829600	0.52079500	-0.54197000
F	-3.55647900	2.17546400	1.39399300
F	-1.81491300	3.39944000	-0.13490900
F	-4.18971100	3.02567500	-0.93310200
F	-0.36823900	1.85655900	2.84872100
F	-1.76957300	-0.39418100	3.17022700
F	-0.38608500	0.58703600	5.05514500
F	1.77935400	0.61509200	3.73619900
F	0.40339700	-1.60410100	4.03218200

HS − *TBPY*-5

Te	-2.02705100	-2.73180900	-1.27385400
Te	1.94698200	2.73353200	-1.37551600
Mn	0.03347800	-0.00771000	-0.25510900
F	-2.35721800	1.68593100	-2.10423600
F	1.70354800	-2.95908800	0.69926200
F	2.53015000	-3.01040500	-1.80115200
F	3.07375200	1.93289200	-0.11293900
F	-2.77101300	-2.25762500	0.37643100
F	2.58692700	1.59233400	-2.71726700
F	-3.40230700	-1.83621600	-2.17877400
F	-1.47320900	-3.44907500	-2.91888600
F	-0.84713400	-3.89409700	-0.39932000
F	1.55003700	4.08133400	-0.13460700
O	1.70801800	-0.64220100	-0.83249100
O	-1.60703900	0.69257100	0.35758100
O	-0.85977900	-1.34500100	-1.38210800
O	0.46060300	1.76067600	-0.98440300
F	1.05835200	3.73271900	-2.69343300
F	-3.22974100	-4.18228800	-1.23085400
F	3.43995200	3.80013000	-1.80508200
O	0.50112300	-0.44441200	1.61193300
Te	2.89177200	-1.93674100	-0.30949000

IS

Te	3.07959500	1.58317000	-0.54246700
Te	-3.18620400	-1.41877200	-0.61167700
Mn	-0.00328800	-0.05479000	-0.44793000
F	0.73413900	-2.29571400	-2.52221200
F	0.06873300	3.41992700	0.12979400
F	-0.83192500	3.66417300	-2.35362200
F	-3.48584100	0.10907400	0.41696300
F	3.73113200	0.01053400	0.22009400
F	-3.44642700	-0.45450900	-2.19586700
F	3.90037600	1.12219300	-2.16480400
F	2.73716000	3.27942800	-1.25598900
F	2.51112200	2.15444000	1.13999100
F	-3.23146000	-2.45768500	0.94458700
O	-1.18830100	1.28329100	-1.15090400
O	1.18543700	-1.49316200	0.05633300
O	1.46253500	0.93197900	-1.10088700
O	-1.35555800	-1.34203500	-0.64074000
F	-3.17932800	-2.99702400	-1.62400600
F	4.75315000	2.31182600	-0.07537700
F	-5.05938700	-1.61396100	-0.61018100
O	-0.19360200	0.54966900	1.28369300
Te	-1.54624400	3.02953700	-0.73287300

Te	1.59822900	-2.94489200	-0.97410900
Te	0.05777300	-0.25150500	2.93218500
F	-1.97166500	4.84099400	-0.42132400
F	-3.23266200	2.95087500	-1.55803600
F	-2.35748100	2.72545500	0.92729600
F	3.21045500	-2.23218400	-1.62077100
F	2.55632200	-3.78515400	0.40046300
F	0.09567500	-3.96660300	-0.51746100
F	2.05695700	-4.43833500	-2.02800700
F	-0.35390900	-2.00878400	2.43997900
F	1.89105600	-0.57870900	2.75086900
F	0.26278700	-0.94220900	4.67273900
F	-1.73706200	0.00305800	3.40880000
F	0.44857700	1.40776100	3.70977400

LS

Te	-1.19430200	-3.09471600	-1.31177400
Te	1.29010000	3.03431100	-1.37082600
Mn	0.14811300	-0.03693400	-0.21808500
F	-2.57037500	1.32043600	-2.22538200
F	2.32326700	-2.37828000	0.97894200
F	3.23267500	-2.38580800	-1.51145500
F	2.56389100	2.67634900	-0.05114800
F	-2.30763500	-2.84468100	0.16575900
F	2.24515100	2.04185100	-2.63931800
F	-2.26895500	-1.99643200	-2.38084700
F	-0.25298200	-3.60824500	-2.85208800
F	-0.27171200	-4.41909200	-0.35978100
F	0.49371100	4.27661500	-0.21808300
O	2.01658700	-0.11390700	-0.63211100
O	-1.74615900	0.03614800	0.07012300
O	0.03544400	-1.80128400	-0.91575200
O	0.10022800	1.71225600	-0.92822800
F	0.17738700	3.65325800	-2.74733500
F	-2.38717500	-4.47284100	-1.78426300
F	2.42333600	4.45523500	-1.86568600
O	0.56279600	-0.01926500	1.63088700
Te	3.35884100	-1.21118700	-0.05315000

Te	-3.13622300	1.10370900	-0.45040500
Te	-0.32842500	0.17086400	3.21861600
F	4.81616900	-2.28725600	0.46840500
F	4.64042000	-0.22714300	-1.00748600
F	3.76550700	-0.20793600	1.47571700
F	-4.18658800	-0.35591000	-0.97478700
F	-3.96426200	1.01546100	1.22667100
F	-2.38843900	2.74943000	0.02745500
F	-4.62959000	2.13329600	-0.96696100
F	-1.39219000	1.61500100	2.68883200
F	-1.70265500	-1.04148700	2.85305800
F	-1.15506200	0.36852100	4.89869900
F	0.93994300	1.38450900	3.87903100
F	0.64132900	-1.21409800	4.02925100

M06-L

HS − *SPY*-5

Te	-3.40767400	0.00410700	-0.82293500
Te	3.47869400	0.03231800	-0.68145000
Mn	0.03769500	0.01806500	-0.80570000
F	1.49574300	3.48886000	-1.78869100
F	-1.06157400	-2.89171500	0.67247500
F	-1.40916800	-3.40856500	-1.91204200
F	2.95052000	-1.05889500	0.73234000
F	-2.93766600	1.02140500	0.66439700
F	3.48203500	-1.43476400	-1.84434800
F	-3.34926300	1.52575200	-1.91140900
F	-4.14112500	-0.93607800	-2.26969300
F	-3.79947400	-1.45811100	0.26782100
F	3.80529800	1.43732700	0.50293400
O	0.66628200	-1.75475200	-1.17417900
O	-0.57855600	1.79915400	-1.14880500
O	-1.72910200	-0.59928900	-1.22582800
O	1.81946400	0.64531700	-1.14470900
F	4.27723000	1.04876100	-2.03948300
F	-5.17493500	0.53448000	-0.46839000
F	5.23133400	-0.50628800	-0.26991600
O	0.02192000	0.00569700	1.17591400

Te	0.04348200	-3.41728300	-0.73133900
Te	0.01175200	3.45629300	-0.64784500
Te	-0.12980600	-0.07685300	2.96777800
F	-0.50530100	-5.17058100	-0.33899100
F	1.07323900	-4.20614100	-2.08476800
F	1.43452600	-3.75664100	0.46475800
F	-0.99113500	4.26577800	-2.00899000
F	-1.41287900	3.74877900	0.52056900
F	1.08957200	2.91589000	0.77113800
F	0.52809500	5.20721000	-0.20201500
F	-0.90045200	1.62337100	3.15800200
F	-1.84426400	-0.83715900	2.97624700
F	-0.27651600	-0.15995800	4.84793200
F	1.55710400	0.67048900	3.31219100
F	0.61708000	-1.79063300	3.12621100

Te	0.13996100	-3.42706600	-0.65815700
Te	-0.06877500	3.36916300	-0.92852300
Te	-0.09952300	0.23812100	3.07548300
F	-0.29725100	-5.23465200	-0.38916600
F	1.68557900	-4.08653200	-1.48073600
F	0.99273400	-3.54295900	0.99496300
F	-1.71931600	3.51877200	-1.79955900
F	-0.75405500	4.36851600	0.49119300
F	1.64084000	3.53148800	-0.20141900
F	0.30594600	5.02327600	-1.73797300
F	1.08453300	1.60473700	2.57383700
F	-1.55338300	1.38605300	2.78732600
F	-0.03390700	1.03472100	4.78252300
F	1.35597000	-0.77562400	3.69305400
F	-1.25858000	-0.99575700	3.88885100

HS − *SPY*-5’

Te	-3.47187200	-0.20228000	-0.76404200
Te	3.49944000	0.00946300	-0.65841900
Mn	0.01697100	-0.00089900	-0.46833100
F	0.63065600	2.59999900	-2.48953700
F	-1.47189500	-3.04860600	0.19684700
F	-0.76757300	-3.62386500	-2.28489700
F	3.17477100	-1.41994600	0.49591700
F	-3.37599200	1.53092600	-0.08229700
F	3.31695800	-1.11072900	-2.14634400
F	-3.90390600	0.50419900	-2.44671500
F	-3.90674400	-1.90223600	-1.41480400
F	-3.33684800	-0.88268300	0.96649900
F	4.00326600	1.06219000	0.79955900
O	0.65127800	-1.70982600	-1.02323700
O	-0.51171100	1.79573900	-0.11515100
O	-1.71564000	-0.40520900	-1.21848000
O	1.80345300	0.67409900	-0.69389200
F	4.11030400	1.36424400	-1.79923700
F	-5.31512100	-0.06504100	-0.41490500
F	5.28704800	-0.57250900	-0.65002300
O	-0.15620400	-0.57157900	1.45849500

HS − *TBPY*-5

Te	-2.48984700	-2.67694000	-0.61116000
Te	2.05001500	2.17235300	-1.81326100
Mn	-0.00884800	-0.16554300	-0.28068800
F	-2.50012500	1.50912300	-2.15482300
F	1.23533100	-3.02862600	1.01467100
F	1.86096400	-3.62793900	-1.47585300
F	3.02288200	1.47377800	-0.37517300
F	-2.71571400	-1.99581700	1.11671800
F	2.64828300	0.79365100	-2.93064500
F	-3.78344800	-1.50876600	-1.28913000
F	-2.48286700	-3.55498300	-2.26910900
F	-1.46623200	-4.08407200	0.07972100
F	1.73620300	3.72674000	-0.82329400
O	1.57291300	-1.01922500	-0.86190300
O	-1.55676500	0.77806100	0.31176500
O	-1.07359000	-1.63282700	-1.05696800
O	0.46127200	1.39645800	-1.37958400
F	1.31987000	3.02869100	-3.31379800
F	-3.95776400	-3.78318700	-0.20770700
F	3.65584600	3.02031800	-2.30463200
O	0.58722200	-0.26490300	1.60599800

Te	2.52312100	-2.43496500	-0.19584400
Te	-2.63759300	2.08639400	-0.38093200
Te	0.55964700	0.86712900	3.02403000
F	3.55895300	-3.89111700	0.38129100
F	3.94973300	-2.05297600	-1.34144400
F	3.38701400	-1.48546300	1.15580600
F	-4.17411200	1.05411400	-0.13879400
F	-2.95799000	2.83550700	1.29879100
F	-1.34653700	3.39903200	-0.66878400
F	-3.83588500	3.37569100	-1.04353300
F	0.31125300	2.43107900	2.02255200
F	-1.27857400	0.73733500	3.34691100
F	0.56407500	2.00462400	4.52312200
F	2.40742100	1.17284600	3.00697700
F	0.84307900	-0.51051300	4.26520800

Te	1.08429000	-3.29786800	-0.49931300
Te	-1.03209200	3.11925500	-1.05268800
Te	-0.11904300	0.53716500	2.88219100
F	1.18444100	-5.13421200	-0.09170400
F	2.76231800	-3.55054300	-1.30495100
F	1.92603100	-3.04870800	1.15521500
F	-2.71594200	2.76131700	-1.80639700
F	-1.88226900	4.03967300	0.33948800
F	0.61445900	3.80313900	-0.47964400
F	-1.15502100	4.73121200	-2.01877600
F	0.50811000	2.18726300	2.23156600
F	-1.90570500	1.06261700	2.61386900
F	-0.26488800	1.40559600	4.55057400
F	1.63652700	0.11751700	3.41946000
F	-0.72009300	-0.99561500	3.79557200

IS

Te	-3.26713100	-1.21054900	-0.60364700
Te	3.33324200	0.86569600	-0.65543000
Mn	0.01681700	-0.03233600	-0.45198400
F	-0.23572600	2.40649900	-2.60663800
F	-0.57658800	-3.34040100	0.36548800
F	0.26993100	-3.87823000	-2.09201800
F	3.37484900	-0.57947600	0.52692900
F	-3.66400900	0.48512300	0.05843600
F	3.47209300	-0.28044900	-2.12989000
F	-3.91884000	-0.69431300	-2.28263200
F	-3.19772400	-2.97217400	-1.22755400
F	-2.88542400	-1.78737000	1.12748600
F	3.51591900	2.03853100	0.79013600
O	1.05361500	-1.53270400	-1.00839500
O	-0.99359200	1.55464700	-0.09628900
O	-1.53350800	-0.89624500	-1.10474900
O	1.50603200	1.07078300	-0.73449100
F	3.56716200	2.31810600	-1.81615200
F	-5.05892600	-1.61409200	-0.20073500
F	5.21147200	0.78158800	-0.62066900
O	0.07473900	-0.47990000	1.31062900

LS

Te	3.08771700	1.53640100	-0.63869200
Te	-3.17889700	-1.38827500	-0.62399600
Mn	-0.01177800	-0.02244600	-0.47761400
F	0.62749500	-2.38462300	-2.46478500
F	0.10593700	3.35748000	0.19572000
F	-0.82287500	3.72038000	-2.25815200
F	-3.46105700	0.11742200	0.44169100
F	3.71047400	-0.05547800	0.10743800
F	-3.44872400	-0.38545400	-2.18112900
F	3.86073300	1.05473600	-2.27745100
F	2.78004700	3.24259800	-1.34386600
F	2.56778100	2.11623900	1.05691800
F	-3.22092200	-2.46326400	0.90576700
O	-1.22301600	1.30747500	-1.14909100
O	1.17525000	-1.45146100	0.05664200
O	1.43211300	0.94476900	-1.17592100
O	-1.34440700	-1.32588400	-0.65314200
F	-3.17339100	-2.94037800	-1.67263500
F	4.78705700	2.21743300	-0.20736100
F	-5.04899100	-1.58077600	-0.61726200
O	-0.18277500	0.58296700	1.24412600

Te	-1.53400800	3.04452200	-0.65368200
Te	1.50269000	-2.97503100	-0.90008200
Te	0.12692000	-0.22583800	2.89222000
F	-1.90984200	4.85254200	-0.28104200
F	-3.23317800	3.02769900	-1.45466600
F	-2.32535400	2.71328200	1.01132800
F	3.12027800	-2.35972400	-1.62978000
F	2.46321200	-3.76220700	0.50159500
F	-0.02373600	-3.91596100	-0.35773400
F	1.88751200	-4.54156800	-1.86984700
F	-0.25542400	-1.99889800	2.41857000
F	1.96776900	-0.51314900	2.68541300
F	0.36699500	-0.89601500	4.63446000
F	-1.67183700	-0.01265100	3.38022700
F	0.48416600	1.44874500	3.65652800

O	0.03576700	0.02488700	1.23104500
Te	2.46207900	-2.36917300	-0.81932600
Te	-2.47399600	2.46370300	-0.57577100
Te	0.10282500	-0.21037100	3.00057000
F	3.40016800	-3.95067600	-0.50494900
F	3.61290500	-2.15412700	-2.26502500
F	3.73489000	-1.57699500	0.26777200
F	-3.69842800	2.32221800	-1.96890200
F	-3.70282400	1.63948500	0.53772400
F	-1.39526600	2.84844000	0.87466200
F	-3.37807400	4.03925400	-0.14859600
F	-1.62889400	0.39627600	3.31307500
F	-0.52779500	-1.96061500	2.97571500
F	0.16996300	-0.44581300	4.85769000
F	0.74171800	1.49861800	3.36970700
F	1.85026000	-0.84888400	3.02939400

M06

HS − *SPY*-5

Te	-2.48303700	-2.39337200	-0.80473900
Te	2.39612900	2.50677700	-0.71975200
Mn	-0.01951700	0.03616600	-0.76019100
F	-1.43162200	3.54808100	-1.66377200
F	1.45826100	-2.82167300	0.66388100
F	1.37341500	-3.41750000	-1.89780400
F	2.88527700	1.41276500	0.68689100
F	-2.92118600	-1.37930000	0.67664800
F	3.42467100	1.49147200	-1.88606100
F	-3.47787200	-1.27123200	-1.89959900
F	-2.28440800	-3.54805000	-2.24986400
F	-1.76232300	-3.70297200	0.28833100
F	1.62077100	3.70190100	0.46413900
O	1.63433600	-0.79908300	-1.18830500
O	-1.68691000	0.90076900	-1.05191000
O	-0.87644300	-1.62164100	-1.13857200
O	0.81976800	1.70563000	-1.11699900
F	2.15503500	3.75003300	-2.08218300
F	-4.10347100	-3.27285900	-0.51766100
F	3.98125100	3.42859300	-0.37547900

HS − *SPY*-5’

Te	-3.39723300	-0.69761800	-0.53642600
Te	3.45290000	0.58115100	-0.55158900
Mn	0.01457200	0.05099800	-0.37906300
F	0.08693400	2.39819500	-2.57792000
F	-0.91803600	-3.30437500	0.03702100
F	-0.31727100	-3.39702200	-2.52373800
F	3.38681100	-0.98516000	0.43115400
F	-3.52602700	0.98621200	0.21906100
F	3.42648900	-0.38018900	-2.13939900
F	-3.97974600	-0.00385500	-2.15939500
F	-3.59551600	-2.39902600	-1.25097400
F	-3.10275000	-1.42367500	1.13881000
F	3.79337400	1.53008900	1.00388800
O	0.87543600	-1.50480300	-1.03938400
O	-0.75579700	1.76812400	-0.07594100
O	-1.65357400	-0.61543600	-1.03003700
O	1.68360300	0.95608900	-0.51902100
F	3.81342600	2.11905000	-1.53107900
F	-5.21303300	-0.84669000	-0.12743900
F	5.29824200	0.30440400	-0.60222800

O	-0.00969000	-0.54799300	1.54777000
Te	0.65090700	-3.30022900	-0.94080700
Te	-0.68807500	3.25141200	-1.11458400
Te	-0.01848200	0.15121200	3.20168800
F	0.50170800	-5.16025100	-0.93024600
F	2.20848500	-3.58494100	-1.91145900
F	1.61337800	-3.51883600	0.62455000
F	-2.35537900	2.96029800	-1.87822800
F	-1.49692800	4.30620800	0.17697600
F	0.96828000	3.83363900	-0.52606400
F	-0.66364200	4.78856100	-2.17018200
F	0.83735200	1.74354600	2.75966800
F	-1.67073000	0.99318500	3.05137000
F	-0.02400100	0.83483200	4.94436600
F	1.62578000	-0.58055400	3.68356000
F	-0.86220000	-1.32375900	3.96667900

HS − *TBPY*-5

Te	-2.59081200	-2.59322500	-0.54528200
Te	2.10787000	2.06933500	-1.85768200
Mn	-0.02172800	-0.15791000	-0.23664100
F	-2.47729600	1.52569100	-2.15186400
F	1.23072500	-3.08131200	1.09758000
F	1.66060700	-3.69843000	-1.41002200
F	3.09419600	1.38173900	-0.44290900
F	-2.90662400	-1.83642400	1.11947000
F	2.65014500	0.67074200	-2.95470100
F	-3.82983200	-1.46022500	-1.33643600
F	-2.50253600	-3.54293300	-2.14349900
F	-1.61708000	-3.94967100	0.26729400
F	1.83907000	3.63101000	-0.89205000
O	1.50140200	-1.10011100	-0.78863100
O	-1.50473300	0.86996900	0.31039500
O	-1.16018100	-1.58096300	-0.96442200
O	0.53065400	1.33313300	-1.37767300
F	1.36692300	2.90593400	-3.34622100
F	-4.07265200	-3.66799900	-0.17204600
F	3.70558500	2.87534500	-2.39280100

O	0.56522200	-0.23018300	1.64276700
Te	2.43893600	-2.53434000	-0.19165600
Te	-2.59023800	2.13943600	-0.40503900
Te	0.63839400	0.93222200	3.01831800
F	3.45809500	-4.00885300	0.32268000
F	3.79116500	-2.20462400	-1.41763000
F	3.40600700	-1.61080700	1.08720500
F	-4.10917000	1.11284700	-0.13019200
F	-2.88321900	2.92597900	1.24692500
F	-1.29763600	3.41762300	-0.74519000
F	-3.78245400	3.40944300	-1.07531100
F	0.42320800	2.46400400	1.98860100
F	-1.17769600	0.89653200	3.40226800
F	0.74857900	2.10152300	4.47080300
F	2.48161500	1.14511000	2.92483400
F	0.89677700	-0.41171100	4.27945900

IS

Te	-3.34953500	-1.05885300	-0.52666300
Te	3.33046900	0.84489200	-0.68299900
Mn	-0.02144400	0.01048700	-0.41925000
F	-0.26894100	2.39394600	-2.54237900
F	-0.62609000	-3.43546900	0.26411600
F	0.09183800	-3.75426600	-2.24214500
F	3.42828800	-0.64886500	0.40464400
F	-3.70920200	0.61882700	0.16559500
F	3.41420100	-0.21491600	-2.20434900
F	-4.02389100	-0.51275000	-2.17124200
F	-3.32018800	-2.80194000	-1.16442900
F	-2.95698900	-1.65462900	1.18176200
F	3.55509700	1.92635200	0.80773700
O	0.93967000	-1.53069800	-1.02532100
O	-0.94962900	1.64585500	-0.01131000
O	-1.63870800	-0.75329600	-1.04523000
O	1.52474100	1.03098900	-0.67299900
F	3.52005300	2.34547200	-1.76303300
F	-5.12785200	-1.43721200	-0.09845100
F	5.19500900	0.77059900	-0.71994500

O	0.03661200	-0.53706800	1.35174700
Te	0.97569000	-3.30677900	-0.66339100
Te	-1.00175400	3.16183500	-1.00443200
Te	0.04824400	0.36322500	2.94328000
F	1.09000500	-5.15615500	-0.41199200
F	2.60038200	-3.50168400	-1.55148900
F	1.88455600	-3.19621100	0.95255500
F	-2.69341000	2.82220200	-1.70207200
F	-1.79225200	4.14047400	0.36198400
F	0.65419200	3.82762800	-0.49231400
F	-1.11085400	4.73172100	-2.01059700
F	0.79279900	1.95011500	2.34092400
F	-1.66079600	1.06895200	2.81333200
F	0.08311300	1.15544500	4.63276300
F	1.75157500	-0.24740400	3.36076700
F	-0.65145000	-1.11612100	3.81990500

LS

Te	-1.43561800	-3.24231800	-0.64969200
Te	1.55697100	2.78125900	-1.41855000
Mn	0.11079400	-0.13377500	-0.23723500
F	-2.41690200	0.98957000	-2.58260800
F	2.23521500	-2.54669500	1.10891200
F	2.87183400	-2.84164400	-1.42739400
F	2.75252600	2.32496500	-0.07938100
F	-2.51040500	-2.59291800	0.70984700
F	2.45969900	1.71957300	-2.64531000
F	-2.44319700	-2.37426100	-1.94369300
F	-0.54430000	-4.14771700	-2.00625600
F	-0.59674200	-4.35239500	0.57891700
F	0.84497200	4.07843600	-0.30008500
O	1.94569000	-0.42237900	-0.65493500
O	-1.77374200	0.14084500	-0.08139100
O	-0.13941800	-1.97738100	-0.55956900
O	0.26210500	1.57289200	-1.01855000
F	0.54098600	3.47244200	-2.81377800
F	-2.70141600	-4.60592500	-0.79326800
F	2.80207400	4.09219400	-1.88450400

O	0.49421400	0.12802500	1.62087600
Te	3.23307700	-1.57499300	-0.11451000
Te	-3.04906900	1.18210900	-0.84278100
Te	-0.30934700	0.86841700	3.06561300
F	4.63934700	-2.71248700	0.35408200
F	4.47021800	-0.78282700	-1.25466100
F	3.85568100	-0.50203600	1.26633300
F	-4.18879000	-0.26109800	-1.10229300
F	-3.90093100	1.49828700	0.77492500
F	-2.21063200	2.82737700	-0.70023500
F	-4.43866900	2.16712800	-1.61264800
F	-1.18139100	2.22528700	2.15113600
F	-1.83477000	-0.18447800	3.01644900
F	-1.06441900	1.61770800	4.59984700
F	1.10700100	2.02646700	3.39379200
F	0.46707600	-0.35098300	4.23579500

TPSSh

HS − *SPY*-5

Te	3.38560000	-0.80276000	-0.37325500
Te	-3.43865700	0.71798300	-0.46302900
Mn	-0.02769800	0.02376900	-0.23652500
F	-1.62646200	-2.55220200	-2.64196700
F	2.35804000	3.19008500	-0.32045800
F	1.61213900	2.00984900	-2.60241800
F	-2.83626400	2.01578000	0.72734300
F	2.84733500	-2.37406200	0.47101100
F	-3.30330500	1.93781600	-1.87327400
F	2.88515000	-1.47779700	-2.04207700
F	4.21964200	0.62762800	-1.23950100
F	4.16304600	-0.22612000	1.22215600
F	-3.87074200	-0.39870800	0.96567700
O	-0.09731500	1.93861100	-0.44680300
O	0.20068700	-1.81632400	-0.72449300
O	1.86037100	0.16890900	-0.07509700
O	-1.82887200	-0.08035700	-0.82877500
F	-4.31566600	-0.45960600	-1.62075300
F	5.00841800	-1.70715600	-0.66450200

F	-5.14225900	1.46641000	-0.19387800
O	-0.51349300	-0.13873100	1.71919000
Te	0.82872800	3.20107500	-1.38719100
Te	-0.76669700	-3.30392300	-1.16137200
Te	-0.01934300	0.18268700	3.43157100
F	1.71967200	4.54657200	-2.35154400
F	-0.55635500	3.48445000	-2.61365100
F	0.14156000	4.59594700	-0.34978800
F	0.57998700	-3.92323900	-2.30264900
F	-0.02244600	-4.33052300	0.21104500
F	-2.24959400	-2.96931800	-0.08259900
F	-1.66874400	-4.88283600	-1.64150900
F	-0.97388000	-1.28054800	4.12247600
F	1.52821600	-0.87641100	3.39458400
F	0.45341700	0.49284700	5.23073200
F	-1.49016200	1.28190500	3.82095700
F	1.01265900	1.70764400	3.07806000

HS − *SPY*-5’

Te	-0.19944000	-0.48033600	-3.46624000
Te	0.36877200	3.44332400	1.16243500
Mn	0.09564700	0.04838200	0.14851300
F	-3.06177600	1.99126000	-0.58782700
F	2.92328400	-1.83631300	-0.74644600
F	3.79177100	0.52653400	-1.54469800
F	1.47168300	2.69009800	2.47217500
F	-1.25555700	-1.95534400	-2.99846800
F	1.78705900	3.49736600	-0.05097500
F	-1.68937300	0.64742800	-3.32029100
F	0.75486200	0.92831700	-4.25970400
F	1.19124400	-1.64648800	-3.94260800
F	-0.95549300	3.69291500	2.46101000
O	1.91058700	0.50006400	0.41632100
O	-1.73901200	-0.37750400	0.01786300
O	0.46151200	-0.16155800	-1.80914400
O	-0.36893400	1.87708400	0.58660000
F	-0.64702000	4.47572700	-0.02103800
F	-0.82812100	-0.80312400	-5.21341300

F	1.04155500	5.09041400	1.77302100
O	0.47423800	-1.64703000	1.02506500
Te	3.53404200	-0.29980100	0.10841700
Te	-3.39316300	0.37359900	0.27414000
Te	-0.30970700	-3.04345300	1.89160100
F	5.25404800	-1.01063400	-0.15361500
F	4.41782900	1.13157700	0.92293900
F	3.58093800	-1.20698700	1.74075100
F	-4.05292500	-0.32478000	-1.32388500
F	-4.03260000	-1.15014700	1.14235100
F	-3.01311600	1.13571200	1.93580100
F	-5.12848700	1.04953600	0.53035900
F	-1.54049700	-2.01188600	2.85299400
F	-1.59438100	-3.43661200	0.59535100
F	-1.04207100	-4.50325900	2.82487200
F	0.87119000	-2.92868800	3.34199500
F	0.83100500	-4.30832000	1.11771100

HS − *TBPY*-5

Te	-0.21150200	-2.83424300	-2.19093200
Te	0.14154600	3.56506900	-0.92653400
Mn	0.06372500	0.10652700	-0.03724500
F	-2.92383900	0.82391000	-1.83291800
F	2.99043900	-1.79173200	0.44370400
F	3.60677300	-0.78981600	-1.91747400
F	1.63941800	3.61630500	0.19182000
F	-1.24414600	-3.45581100	-0.76155700
F	1.17626800	2.90516300	-2.33827200
F	-1.75974700	-2.33940600	-3.11943800
F	0.77901400	-2.50580800	-3.74732500
F	1.30097400	-3.62607300	-1.42457700
F	-0.82009800	4.52352100	0.36388300
O	1.84570500	0.60042900	-0.39225800
O	-1.72811400	-0.30055100	0.40031100
O	0.09990100	-1.17428700	-1.52668800
O	-0.49869500	1.95881600	-0.35037800
F	-1.27368600	3.82988300	-2.12443500
F	-0.51193800	-4.53466300	-2.94183900

F	0.72552700	5.25758500	-1.50731500
O	0.59306800	-0.47072100	1.77280500
Te	3.51920600	-0.11760000	-0.17734400
Te	-3.40666600	0.21767300	-0.13575300
Te	-0.04746200	-0.83836700	3.43451800
F	5.27642100	-0.75991800	-0.00720300
F	4.31177200	1.45925500	-0.78826800
F	3.73562600	0.48726300	1.57438300
F	-3.81170600	-1.45521600	-0.85678600
F	-4.15252400	-0.35370100	1.47744000
F	-3.32674400	1.94899900	0.55081100
F	-5.16354700	0.67136500	-0.62514100
F	-1.37881000	0.47702100	3.45165200
F	-1.29202000	-2.13250500	2.91326300
F	-0.63223700	-1.22210100	5.18263900
F	1.11700000	0.36723500	4.27495000
F	1.20576200	-2.19900500	3.73413800

F	5.36596100	0.06227400	-0.80075200
O	0.00789200	-0.42144500	1.52468900
Te	0.52105900	-3.46279200	-0.59387000
Te	-0.51420900	3.28005700	-1.09236200
Te	-0.09375700	0.43659900	3.14695500
F	0.31674500	-5.32408200	-0.40479100
F	2.06517600	-3.89746100	-1.56063800
F	1.50856400	-3.57225400	0.98984000
F	-2.21605200	3.09490900	-1.85320600
F	-1.26776900	4.34443200	0.24811400
F	1.18556900	3.78814000	-0.50521900
F	-0.43539500	4.86248400	-2.10761600
F	0.81088800	1.99835000	2.65430000
F	-1.74489000	1.26774500	2.85626000
F	-0.18603800	1.21696200	4.85824400
F	1.52907600	-0.28808600	3.74568800
F	-0.98226500	-1.01186200	3.93968200

IS

Te	-3.43326100	-0.63483200	-0.71950200
Te	3.51593400	0.38152400	-0.68642500
Mn	0.04333000	-0.00229100	-0.33149400
F	0.21324800	2.44440700	-2.60927000
F	-1.05334400	-3.34033500	0.40176400
F	-0.47350500	-3.68398200	-2.16667400
F	3.48046500	-1.11063300	0.43139300
F	-3.62114900	1.06836100	0.01186100
F	3.39834700	-0.70277700	-2.20524800
F	-3.80459400	0.05577400	-2.41948700
F	-3.57983000	-2.35437200	-1.43688600
F	-3.35121000	-1.35433100	0.99891200
F	3.95049300	1.45055700	0.78361000
O	0.79793400	-1.67174500	-0.86476900
O	-0.65229200	1.76282200	-0.06909700
O	-1.62615200	-0.59355400	-1.00963300
O	1.73497900	0.80685800	-0.60992500
F	3.86349000	1.84036200	-1.80493000
F	-5.30108200	-0.74737200	-0.52629200

LS

Te	-0.38542400	-1.78596800	-2.99190200
Te	0.57917600	3.53014100	-0.01282100
Mn	0.13159100	0.04966600	-0.00006900
F	-2.95801700	1.74045500	-1.34083800
F	2.76909000	-2.18729000	0.00983100
F	3.70416100	-0.55737200	-1.86047600
F	1.82364900	3.15513900	1.32870300
F	-1.52844200	-2.81187200	-1.92968000
F	1.82266600	3.14542600	-1.35253500
F	-1.72506100	-0.51902900	-3.29477300
F	0.66143700	-0.94424600	-4.29697500
F	0.85976900	-3.18255200	-2.95185100
F	-0.56902200	4.22050100	1.29323400
O	1.99705300	0.49527900	-0.00133800
O	-1.73774700	-0.36486900	0.00101900
O	0.44173200	-0.88402200	-1.63038500
O	-0.26962600	1.90182800	-0.00671400
F	-0.56990400	4.21114500	-1.32300400
F	-1.14867500	-2.71528100	-4.43764600

F	1.34323900	5.24910100	-0.01931800	F	-2.96010500	1.75112500	1.32430800
O	0.44071100	-0.87125000	1.63769400	F	-5.06336400	1.28881600	-0.00804600
Te	3.54170300	-0.48522700	0.00288900	F	-1.72725400	-0.49105100	3.29701900
Te	-3.35482200	0.49716400	-0.00356900	F	-1.53160800	-2.79483400	1.95014900
Te	-0.38866700	-1.76172900	3.00543200	F	-1.15433900	-2.67883600	4.45766300
F	5.18734800	-1.39867300	0.00687600	F	0.65780000	-0.91085200	4.30488500
F	4.58628700	1.06840700	-0.00329300	F	0.85516000	-3.15984600	2.97771000
F	3.70362300	-0.54206000	1.86683500				
F	-4.06544800	-0.64920600	-1.29821900				
F	-4.06731700	-0.63880300	1.29921700				

$[MnF_5]^{2-}$

B3LYP

SPY-5

Mn	0.00003800	0.00006900	-0.08244300
F	-0.01330500	1.84723400	-0.44328900
F	0.01371800	-1.84755200	-0.44232400
F	1.84911100	0.01320100	-0.40573100
F	-1.84877000	-0.01380000	-0.40679900
F	-0.00086000	0.00072700	1.92715100

TBPY-5

Mn	0.00000500	-0.00126100	-0.00029700
F	-1.84511300	-0.00338500	-0.00073000
F	1.84514500	-0.00350000	-0.00075900
F	0.00003300	0.86028900	1.75256000
F	0.00001100	1.09825300	-1.61418500
F	-0.00008900	-1.94815400	-0.13606200

TBPY-5

Mn	-0.00011200	0.00193100	0.00077600
F	-1.85052300	0.00231900	0.00131700
F	1.85035100	0.00263900	0.00160200
F	0.00039900	-0.67255900	-1.83396300
F	0.00015200	-1.25989000	1.49239100
F	-0.00006800	1.92212600	0.33649700

M06

SPY-5

Mn	-0.00000400	0.00011500	-0.08412700
F	-0.13310100	1.83241900	-0.44089600
F	0.13443800	-1.83284800	-0.43917200
F	1.83395300	0.13314700	-0.39701900
F	-1.83351700	-0.13423300	-0.39914300
F	-0.00176200	0.00119500	1.90991600

M06-L

SPY-5

Mn	-0.00001500	0.00000900	-0.08747100
F	-0.61030300	1.74725100	-0.44555900
F	0.61219200	-1.74894400	-0.44019300
F	1.74966700	0.60986800	-0.39345400
F	-1.74892900	-0.61179500	-0.39505500
F	-0.00258600	0.00359600	1.91723700

TBPY-5

Mn	-0.00002400	0.00141600	0.00053000
F	-1.83547000	0.00286500	0.00133000
F	1.83540300	0.00294600	0.00145500
F	0.00013600	-0.70449600	-1.80507800
F	0.00001500	-1.21993200	1.50566600
F	-0.00001800	1.91468600	0.29515500

TPSSh

SPY-5

Mn	0.00004700	0.00009700	-0.08709800
F	-0.03067900	1.84373100	-0.43705600
F	0.03120900	-1.84397100	-0.43632800
F	1.84536000	0.03064600	-0.40181600
F	-1.84499700	-0.03119800	-0.40308000
F	-0.00102300	0.00052000	1.92021900

TBPY-5

Mn	-0.00000200	-0.00155000	-0.00037900
F	-1.84175800	-0.00368400	-0.00093200
F	1.84177600	-0.00375400	-0.00097900
F	0.00004200	0.84329800	1.75685300
F	-0.00000300	1.11153400	-1.60069600
F	-0.00005000	-1.94308700	-0.15319300

7. Bond Valence Sum analysis

To further determine the oxidation state of the Mn centers in both the $[Mn(OTeF_5)_4]^{2-}$ and $[Mn(OTeF_5)_5]^{2-}$ anions, the Bond Valence Sum (BVS) method was employed.[27] According to this analysis, the sum of the valences of the individual bonds s_{ij} surrounding the *j*th atom is equal to its oxidation state z_j:

$$z_j = \sum_i s_{ij}$$

$$s_{ij} = \exp\left[\frac{R_0 - r_{ij}}{b}\right]$$

where r_{ij} is the observed bond length, R_0 is a constant that is dependent on the nature of the *ij* pair and b = 0.37.

Whereas for **1*** the crystal structure has a sufficient quality (see Table S1), for **2** the data set is not good enough to precisely determine bond lengths (see Table S2). Nevertheless, as for the $[Mn(OTeF_5)_4]^{2-}$ anion we have proved that there is a very good agreement between the experimental and the calculated structures, we have performed the Bond Valence Sum analysis with the calculated minimum of the $[Mn(OTeF_5)_5]^{2-}$ anion (as it appears in Figure 3a). The results of these analyses appear in Table S14.

Table S14. Results of the BVS analysis for the Mn atom in the $[Mn(OTeF_5)_4]^{2-}$ and $[Mn(OTeF_5)_5]^{2-}$ anions.

	R_0[a] [Å]	z_j	**Assignment**
**$[Mn(OTeF_5)_4]^{2-}$**	1.754	1.94	+II
**$[Mn(OTeF_5)_5]^{2-}$**	1.754	3.14	+III

[a] Value taken from Ref. 27 to calculate the oxidation state of the Mn atom in Mn complexes with O donor atoms with no prior assumptions.

8. References

1. N. S. Gill, F. B. Taylor, W. E. Hatfield, W. E. Parker, C. S. Fountain and F. L. Bunger, Tetrahalo Complexes of Dipositive Metals in the First Transition Series, *Inorg. Synth.*, 1967, **IX**, 136-142.
2. M. R. Colsman, T. D. Newbound, L. J. Marshall, M. D. Noirot, M. M. Miller, G. P. Wulfsberg, J. S. Frye, O. P. Anderson and S. H. Strauss, Silver(I) Complexes of Dichloromethane and 1,2-Dichloroethane, *J. Am. Chem. Soc.*, 1990, **112**, 2349-2362.
3. S. H. Strauss, M. D. Noirot and O. P. Anderson, Preparation and Characterization of Silver(I) Teflate Complexes: Bridging $OTeF_5$ Groups in the Solid State and in Solution, *Inorg. Chem.*, 1985, **24**, 4307-4311.
4. C. J. Schack and K. O. Christe, New syntheses of pentafluorotellurium hypochlorite, *J. Fluorine Chem.*, 1982, **21**, 393-396.
5. S. Stoll and A. Schweiger, EasySpin, a comprehensive software package for spectral simulation and analysis in EPR, *J. Magn. Reson.*, 2006, **178**, 42-55.
6. G. Sheldrick, Structure determination revisited, *Acta Crystallogr. A*, 2015, **71**, s9.
7. G. Sheldrick, Crystal structure refinement with *SHELXL*, *Acta Crystallogr. C*, 2015, **71**, 3-8.
8. O. V. Dolomanov, L. J. Bourhis, R. J. Gildea, J. A. K. Howard and H. Puschmann, *OLEX2*: a complete structure solution, refinement and analysis program, *J. Appl. Crystallogr.*, 2009, **42**, 339-341.
9. K. Brandenburg, *DIAMOND*, Crystal Impact GbR, Bonn, 2014.
10. G. A. Bain and J. F. Berry, Diamagnetic Corrections and Pascal's Constants, *J. Chem. Educ.*, 2008, **85**, 532-536.
11. N. F. Chilton, R. P. Anderson, L. D. Turner, A. Soncini and K. S. Murray, PHI: A Powerful New Program for the Analysis of Anisotropic Monomeric and Exchange-Coupled Polynuclear *d*- and *f*-Block Complexes, *J. Comput. Chem.*, 2013, **34**, 1164-1175.
12. M. J. Frisch, G. W. Trucks, H. B. Schlegel, G. E. Scuseria, M. A. Robb, J. R. Cheeseman, G. Scalmani, V. Barone, G. A. Petersson, H. Nakatsuji, X. Li, M. Caricato, A. V. Marenich, J. Bloino, B. G. Janesko, R. Gomperts, B. Mennucci, H. P. Hratchian, J. V. Ortiz, A. F. Izmaylov, J. L. Sonnenberg, Williams, F. Ding, F. Lipparini, F. Egidi, J. Goings, B. Peng, A. Petrone, T. Henderson, D. Ranasinghe, V. G. Zakrzewski, J. Gao, N. Rega, G. Zheng, W. Liang, M. Hada, M. Ehara, K. Toyota, R. Fukuda, J. Hasegawa, M. Ishida, T. Nakajima, Y. Honda, O. Kitao, H. Nakai, T. Vreven, K. Throssell, J. A. Montgomery Jr., J. E. Peralta, F. Ogliaro, M. J. Bearpark, J. J. Heyd, E. N. Brothers, K. N. Kudin, V. N. Staroverov, T. A. Keith, R. Kobayashi, J. Normand, K. Raghavachari, A. P. Rendell, J. C. Burant, S. S. Iyengar, J. Tomasi, M. Cossi, J. M. Millam, M. Klene, C. Adamo, R. Cammi, J. W. Ochterski, R. L. Martin, K. Morokuma, O. Farkas, J. B. Foresman and D. J. Fox, *Gaussian 16 (Revision C.01)*, Gaussian, Inc., Wallingford CT, 2016.
13. A. D. Becke, A new mixing of Hartree–Fock and local density-functional theories, *J. Chem. Phys.*, 1993, **98**, 1372-1377.
14. S. Grimme, J. Antony, S. Ehrlich and H. Krieg, A consistent and accurate *ab initio* parametrization of density functional dispersion correction (DFT-D) for the 94 elements H-Pu, *J. Chem. Phys.*, 2010, **132**, 154104.
15. E. R. Johnson and A. D. Becke, A post-Hartree–Fock model of intermolecular interactions, *J. Chem. Phys.*, 2005, **123**, 024101.
16. J. Tao, J. P. Perdew, V. N. Staroverov and G. E. Scuseria, Climbing the Density Functional Ladder: Nonempirical Meta–Generalized Gradient Approximation Designed for Molecules and Solids, *Phys. Rev. Lett.*, 2003, **91**, 146401.
17. V. N. Staroverov, G. E. Scuseria, J. Tao and J. P. Perdew, Comparative assessment of a new nonempirical density functional: Molecules and hydrogen-bonded complexes, *J. Chem. Phys.*, 2003, **119**, 12129-12137.

18. V. N. Staroverov, G. E. Scuseria, J. Tao and J. P. Perdew, Erratum: “Comparative assessment of a new nonempirical density functional: Molecules and hydrogen-bonded complexes” [J. Chem. Phys. 119, 12129 (2003)], *J. Chem. Phys.*, 2004, **121**, 11507.
19. Y. Zhao and D. G. Truhlar, The M06 suite of density functionals for main group thermochemistry, thermochemical kinetics, noncovalent interactions, excited states, and transition elements: two new functionals and systematic testing of four M06-class functionals and 12 other functionals, *Theor. Chem. Acc.*, 2008, **120**, 215-241.
20. Y. Zhao and D. G. Truhlar, A new local density functional for main-group thermochemistry, transition metal bonding, thermochemical kinetics, and noncovalent interactions, *J. Chem. Phys.*, 2006, **125**, 194101.
21. F. Weigend and R. Ahlrichs, Balanced basis sets of split valence, triple zeta valence and quadruple zeta valence quality for H to Rn: Design and assessment of accuracy, *Phys. Chem. Chem. Phys.*, 2005, **7**, 3297-3305.
22. F. Neese, Software update: The ORCA program system—Version 5.0, *WIREs Comput. Mol. Sci.*, 2022, **12**, e1606.
23. F. Neese, The ORCA program system, *WIREs Comput. Mol. Sci.*, 2012, **2**, 73-78.
24. F. Weigend, Hartree–Fock exchange fitting basis sets for H to Rn, *J. Comput. Chem.*, 2008, **29**, 167-175.
25. A. Martín Pendás and E. Francisco, *Promolden: A QTAIM/IQA code* (available from the authors upon request).
26. K. Momma and F. Izumi, *VESTA 3* for three-dimensional visualization of crystal, volumetric and morphology data, *J. Appl. Crystallogr.*, 2011, **44**, 1272-1276.
27. G. S. Palenik, Bond Valence Sums in Coordination Chemistry Using Oxidation State Independent R0 Values. A Simple Method for Calculating the Oxidation State of Manganese in Complexes Containing Only Mn−O Bonds, *Inorg. Chem.*, 1997, **36**, 4888-4890.
